# Preclinical validation and treatment of volumetric modulated arc therapy based total bone marrow irradiation in Halcyon™ ring gantry linear accelerator

**DOI:** 10.1186/s13014-022-02109-z

**Published:** 2022-08-19

**Authors:** Tanweer Shahid, Sourav Mandal, Subhra Snigdha Biswal, Arundhati De, Mukti Mukherjee, Sandipan Roy Chowdhury, Anupam Chakrapani, Kirubha George, Jibak Bhattacharya, Prosenjit Soren, Tanmoy Ghosh, Biplab Sarkar, Luca Cozzi

**Affiliations:** 1 Department of Radiation Oncology, Apollo Multispeciality Hospitals, Kolkata, India; 2 Department of Hemato Oncology, Apollo Multispeciality Hospitals, Kolkata, India; 3 Radiotherapy and Radiosurgery Department, Humanitas Research Hospital and Cancer Center, Via Manzoni 56, 20089 Rozzano, Milan, Italy; 4 Department of Biomedical Sciences, Humanitas University, Via Rita Levi Montalcini 4, 20090 Pieve Emanuele, Milan, Italy; 5 Varian Medical Systems, Palo Alto, USA

**Keywords:** TMI, TMLI, TBI, Halcyon, VMAT

## Abstract

**Aim:**

This study aims​ to report preclinical validation, and the first clinical treatment of total bone marrow irradiation (TMI) and total bone marrow and lymph nodal irradiation (TMLI) using Volumetric modulated arc therapy in Halcyon-E ring gantry linear accelerator. Preclinical validation includes simulation, planning, patient-specific QA, and dry run.

**Material and method:**

Four patients, two female and two male, with body weights of 116 kg, 52 kg, 64 kg, and 62 kg; with two with chronic myeloid leukemia, one each with acute lymphoblastic leukemia and acute myeloid leukemia (AML) were simulated and planned for TMI/TMLI. Patients were immobilized with a full-body vacuum bag. Head first supine (HFS) and Feet first supine (FFS) CT scans were acquired from head to knee and knee to toe. Planning target volume (PTV) was created with a uniform margin of 6 mm over the total bone marrow/bone marrow + lymph nodes. HFS and FFS PTVs were optimized independently using 6MV unflatten energy for 12 Gy in 6 fractions. Plans were merged to create the resultant dose distribution using a junction bias dose matching technique. The total number of isocenters was ≤ 10 per CT set, and two to four full arcs were used for each isocenter. A junction dose gradient technique was used for dose feathering between arcs between adjacent isocenters.

**Result:**

Only one female patient diagnosed as AML received the TMLI treatment, while the other three patients dropped out due to clinical complications and comorbidities that developed in the time between simulation and treatment. The result presented has been averaged over all four patients. For PTV, 95% dose was normalised to 95% volume, PTV_V107% receiving 3.3 ± 3.1%. Total lung mean and V12Gy were 1048.6 ± 107.1 cGy and 19.5 ± 12.1%. Maximum lens doses were 489.5 ± 35.5 cGy (left: L) and 497 ± 69.2 cGy (right: R). The mean cardiac and bilateral kidney doses were 921.75 ± 89.2 cGy, 917.9 ± 63.2 cGy (L), and 805.9 ± 9.7 cGy (R). Average Monitor Unit was 7738.25 ± 1056.6. The median number of isocenters was 17(HFS+FFS), average MU/Dose (cGy) ratio per isocenter was 2.28 ± 0.3.

**Conclusion:**

Halcyon-E ring gantry linear accelerator capable of planning and delivering TMI/TMLI.​​

**Supplementary Information:**

The online version contains supplementary material available at 10.1186/s13014-022-02109-z.

## Introduction

Total body irradiation has been an essential constituent of conditioning to allogeneic haematopoietic stem-cell transplantation (HSCT) for both acute lymphoblastic leukemia acute and myeloid leukemia [1]. The primary aim of total body irradiation (TBI) is to exterminate malignant cells from the circulating blood, bone marrow, and lymph nodes. Unlike chemotherapy kinematics, radiation delivery to leukemic sites is neither dependent on blood supply, metabolism, and biodistribution nor on the inter-patient variability of drug absorption or clearance kinetics; radiation therapy can also reach sites such as the brain or testes, which are often inaccessible for drugs [2]. Total body irradiation can induce effective immunosuppression to avoid the rejection of donor haematopoietic cells [2]. The TBI technique, originally developed by Edward Donnall Thomas in 1975, still remains the same, unable to irradiate the target without exposing healthy structures to the planned dose [3, 4]. TBI is primarily limited by the toxicity to critical organs, especially the lungs, eyes, heart, liver, and kidneys [5–7]. When using total body irradiation with this goal, the need for reducing toxicity through technical optimization and new approaches emerges as total marrow irradiation (TMI) or total bone marrow and lymph nodal irradiation (TMLI) [7]. Although introduced in 2005 TMI is primarily limited to Tomotherapy machines. Intensity-modulated radiotherapy (IMRT) based TMI was introduced by Wilkie et al. in 2008 and Volumetric Modulated Arc Therapy (VMAT) based TMI using C-Arm linear accelerator was presented by Fogliata et al. and Aydogan et al. [8–10]. Total marrow (and lymph-nodes) irradiation (TMI-TMLI) reduced the dose to OARs, maintaining the dose coverage of hematopoietic target or lymphoid tissues. However, this VMAT based technique never gained potential in regular clinical practice [11]. There are many challenges contributing to the slow adoption of the VMAT based TMI/TMLI technique in clinical routine due to limitations in the optimizer engines to simultaneously optimise a large number of fields/arcs in multiple isocentres. The introduction of GPU (graphics processing unit) based systems in radiotherapy planning has largely solved the optimization problem.

From a delivery point of view, recently, Varian launched the 3^rd^ generation (Version E) ring gantry accelerator, Halcyon-E (Varian Medical System, Polo Alto, CA), which offers a high dose rate unflatten beam, fast gantry rotation, high multileaf collimator (MLC) speed, capability of delivery of the volumetric arc along with adjacent field junction dose uniformity and artificial intelligence based volumetric image matching (i-CBCT). These are the essential components of the seamless planning and delivery of TMLI/TMI. This article presents the preclinical validation and delivery of the TMI/TMLI patient in the new ring gantry linear Halcyon accelerator.

## Material and method

Characteristic linear accelerators: Halcyon, a ring gantry linear accelerator with no couch angular motion, source to isocenter distance as 100 cm, have no jaws, and it is equipped with two staggered stacks of 1 cm width Multileaf collimator (MLC) with an effective resolution of 5 mm defining the largest field opening of 28 × 28 cm^2^ and lone flattening filter-free X-ray beam of 6MV with a maximum dose rate and MLC speed of 800 MU/min and 5 cm/s respectively. For VMAT, the gantry rotation speed is two revolutions per minute.

### Simulation

**Fig. 1:**
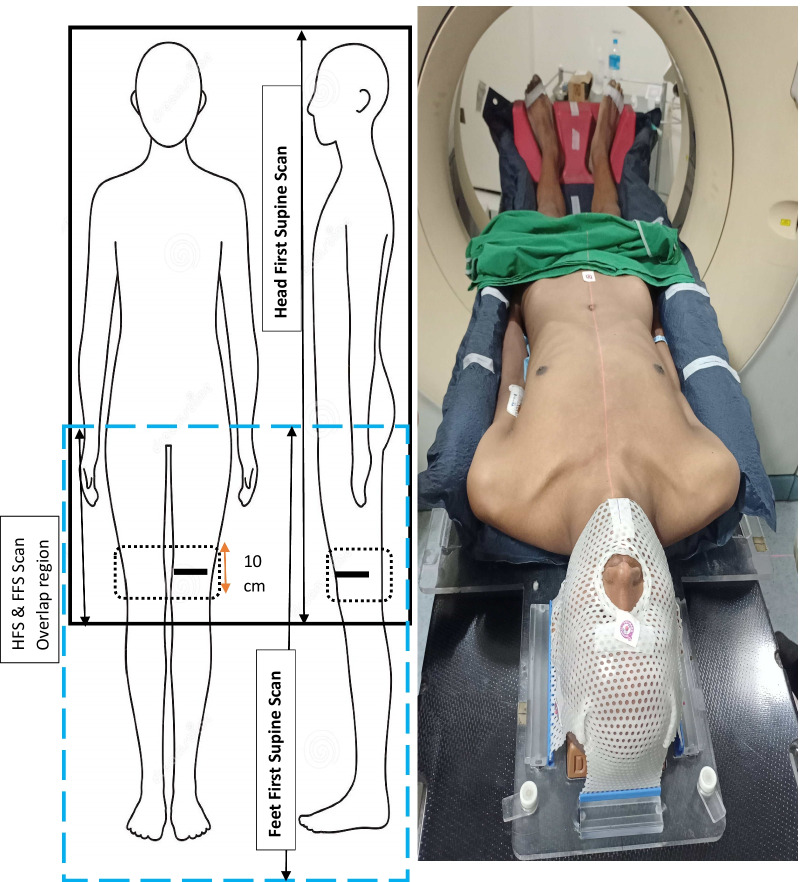
Left panel: Head first supine and feet first supine scanning length. Junction plan in thigh region shown in the sagittal and coronal plane by the dotted box of the craniocaudal length of 10 cm. A solid black line indicates the lead wire's position, which longitudinally bifurcate the box. The right panel shows the patient's FFS simulation and the immobilization devices

Four patients were simulated for TMI treatment in our center. Only pediatric patients can be treated with unidirectional table movement because of the Halcyon table movement (< 144 cm). For the headfirst supine (HFS) adult patients only up to mid-thigh can be treated, leading to a requirement of simulating such patients twice, once in the headfirst supine position-above the head to mid-thigh (at least), again in feet first supine position (FFS) from the pelvis to end of the feet. The patient was placed in a full-body vacuum bag, head, and neck immobilization board, and foot stabilizer, as shown in Fig. 1. Foot stabilizers ensure the positional reproducibility of the lower limb and foot. The patient's head was immobilized with a three clamp thermoplastic. Hand and feet fingers were tied together with adhesive tape and an impression of hand was made on the vacuum bag. Figure 1 shows the simulation limits and actual patient position for the FFS condition. Different axial levels, shoulder, chest, pelvis, thigh, and central sagittal plane of the patients were marked along with the vacuum bag for positional reproducibility. Both HFS and FFS scans were obtained with 5 mm slices acquired on a Philips Big Bore 16 slice CT Scanner (Philips Medical System, Amsterdam, The Netherland). CT Images were pushed to Soma Vision (Varian Medical System, Polo Alto, CA) contouring station.

### Contouring

**Fig. 2:**
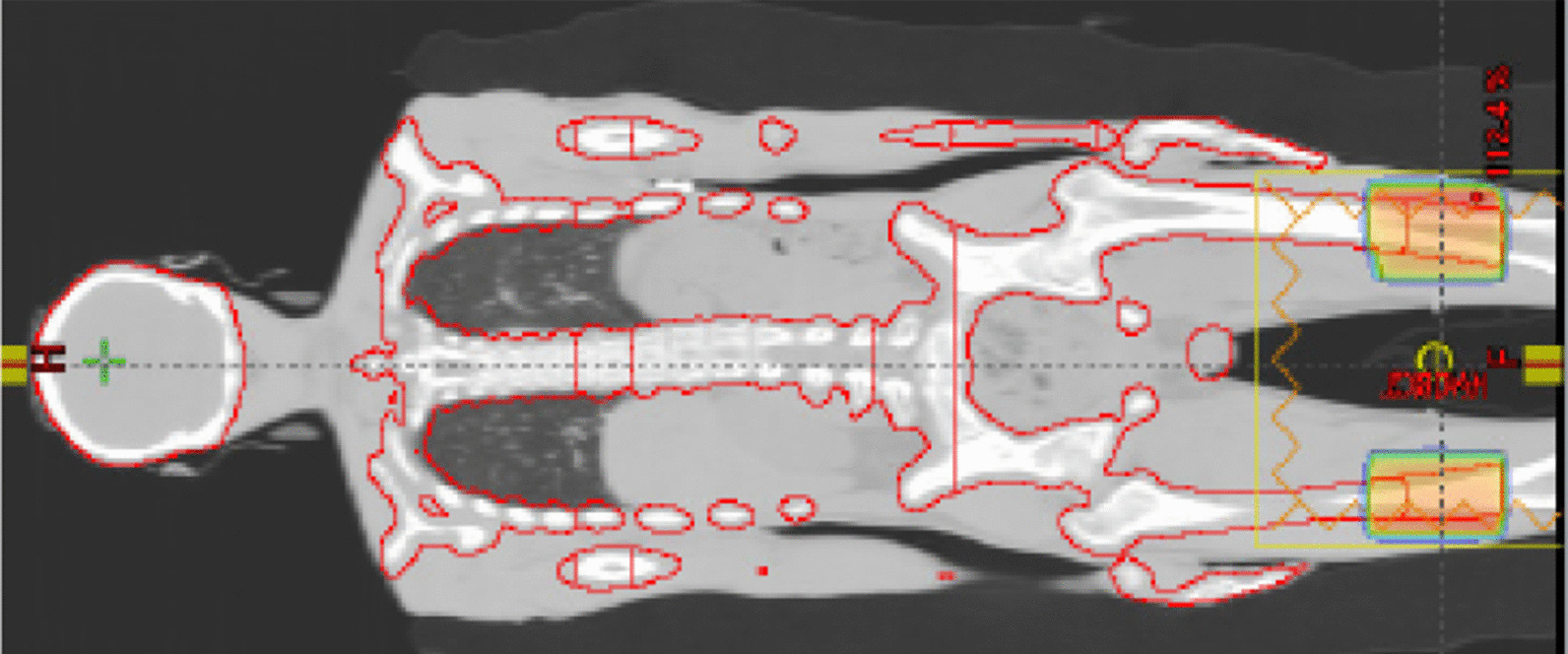
Target volume and junctional plan at thigh level for HFS CT

Total lymph nodal stations and bone marrow was contoured as clinical target volume by two experienced clinicians and peer-reviewed by additional two clinicians independently, one from the institution other from outside. Contouring time is around three to four working days for two clinicians, sequentially doing target and organs at risk (OAR). A 5 mm margin was applied to define the planning target volume (PTV) presented in Fig. 2.

All organs at risk (OAR) were contoured, which include the bladder, bowel, brain stem, optic chiasm, bilateral cochlea, duodenum, oesophagus, bilateral eyes, gland thyroid, gonads, heart, bilateral kidneys, larynx, bilateral lens, whole and bilateral lung, mandible, bilateral optic nerve, oral cavity, bilateral parotid, pituitary, rectum, stomach and testis/ovary.

### Treatment planning and dose constraints

Additional file 1 shows the simulation and planning for HFS and FFS CT Scans and the optimization constraints used for one patient. 6MV FFF beam was used for the planning in the Eclipse treatment planning system (TPS) V15.6 planning system using PO (photon optimizer) and Anisotropic Analytical Algorithm (AAA). For HFS scan, in the inferior-thigh, a junction contour was created against the wire placed during the simulation having a craniocaudal length of 10 cm that matches the boundary with the PTV (Fig. 2). Wire bifurcates the junction contour in the 5 cm cranial and caudal side. This junction contour was then copied to the FFS scan. This makes the junction contour identical in both the scans.

An anterior–posterior beam plan (HFS_JN) with 90° of collimator angle was done for the HFS scan for this junction contour as shown in Fig. 2; this plan was copied to the HFS scan and placed with a 180° collimator rotation (HFS_JN). These two plans will serve as a base plan while doing the VMAT optimization for the rest of the PTVs. The VMAT plan for the HFS and FFS conditions was carried out independently, taking HFS_JN and FFS_JN plans, respectively, as the base dose plan. For ease of VMAT optimization, HFS CT PTV was segmented into five sections abdomen, hand, head, pelvis, and thorax, as shown in the Additional file 1. First, all arcs in each CT set were simultaneously optimised to create a resultant plan. The final dose distribution created merging HFS, FFS, and HFS_JN plan. For treatment delivery, HFS and FFS plans were then split, and a KV Cone Beam CT (CBCT) was attached to each plan for positional verification.

### Isocentre placement strategy

**Fig. 3:**
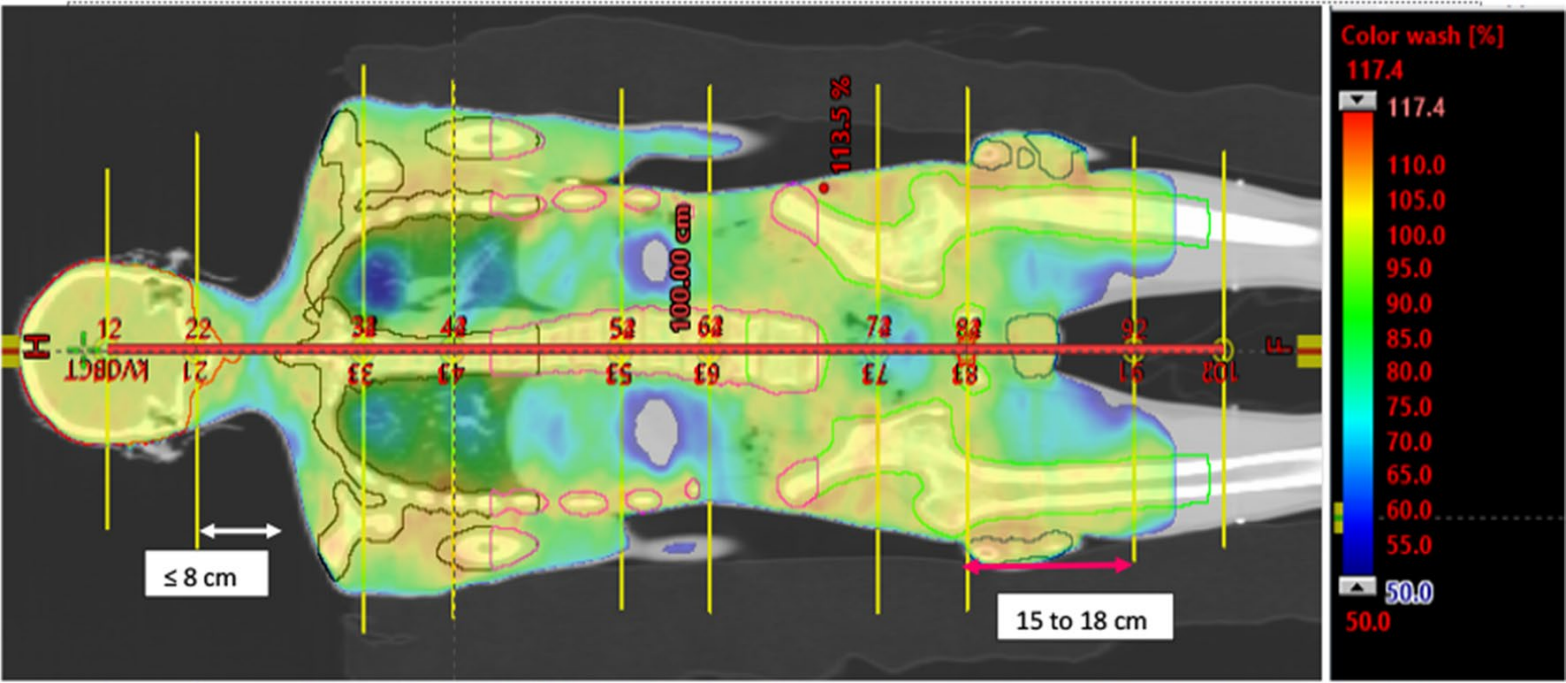
Icocenter and arc placement and dose distribution until 50% of the prescription dose for HFS CT set. Further detail can be found in Additional file 1

The location of the isocenters in the coronal plane of the HFS CT is depicted in Fig. 3. Only one longitudinal shift was permitted between the initial and subsequent isocenters in Halcyon. In the Additional file 1, a detailed view of the arc arrangement is presented. Arcs were given names like {1–1, 1–2}, {2–1, 2–2}, {3–1, 3–2, 3–3, 3–4}, {4–1, 4–2, 4–3, 4–4} etc. The first entity indicates the number of isocenters, and the second entity is the number of arcs. First, isocenter has 2 arcs, 1–1 and 1–2. Isocenter 3 has four arcs: 3–1, 3–2, 3–3, and 3–4 etc.

Adjacent isocenter pairs 1 and 2, 3 and 4, 5 and 6, etc., are placed within 8 cm of each other to avoid acquiring CBCT image in the 2nd isocenter. The gap between the 2nd and 3rd, 4th and 5th, 6th and 7th isocenters varies between 15 and 18 cm depending upon the craniocaudal length of the target volume. As a result, isocenters are not all distributed equally along the patient's long axis. The isocenter placement strategy is based on three criteria: a minimum number of CBCT, an effective low gradient dose build up at the field junction, and an acceptable dose distribution while sparing the OAR. The overlap between adjacent fields should be preferably around 10 cm, which indicates an isocenter separation of 18 cm for a field size of 28 × 28 cm^2^. For a 15 cm separation field overlap, it is 13 cm. This arrangement helps to optimize (minimize) the number of CBCTs, hence on-couch time during therapy delivery.

The number of isocenters and their placement was adopted from the VMAT based craniospinal irradiation technique and further improvised for the TMLI technique [12–14]

Arc per isocenter is not fixed and varies according to the dosimetric requirement. The first two cranial isocenters (covers until mid-neck region) in HFS and all isocenters in FFS have two full arcs; the rest contain two additional partial arcs ( G220 to G140 with avoidance from G300 to G60). Detail of the number of arcs per isocenter and optimization dose constraints are presented in the Additional file 1. Dose constraints considered for initial planning include at least 95% of the target volume receiving 95% of the prescription dose, with 107% of dose not exceeding 5% volume. Mean lung and Kidney dose 10 Gy, Lens maximum dose < 8 Gy for the rest of the organs as low as achievable without compromising PTV dose coverage.

### Patient hospitalisation and clinical investigations

All patients were admitted to the hospital for bone marrow transplants prior to the simulation under the department of hemato-oncology. Patients were found to be in good general health with no other diseases or morbidities during their clinical examination. Ophthalmologic tests, thyroid function test, and endoscopy were carried out before the beginning of BMT. Both the female patients had a complete family, with two children each. The ages of the youngest children were 5.5 years and 9 years, respectively.

### Patient specific QA

Patient-specific quality assurance was performed for the safe delivery of TMI. A MU to dose correspondence was verified using a 0.6 cm^3^ ion chamber (PTW Freiburg, GMBH) placed inside 10 × 30 × 30 cm^3^ solid water phantom by merging all delivered fields per isocenter [15]. Per isocenter portal dose verification was carried out using gamma index method using 2%(DD)–2 mm(DTA), 2%–3 mm, 3%–2 mm and 3%–3 mm dose difference (DD) and distance to agreement (DTA) analysis.

## Result

**Fig. 4:**
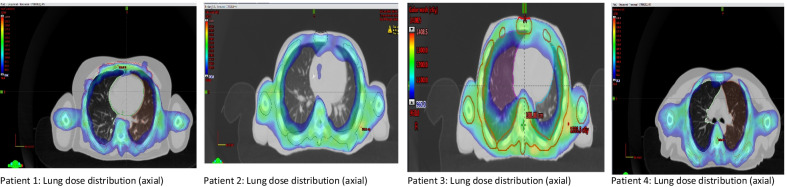
Dose distribution in the axial cut at mid lung level of the four simulated patients

**Fig. 5:**
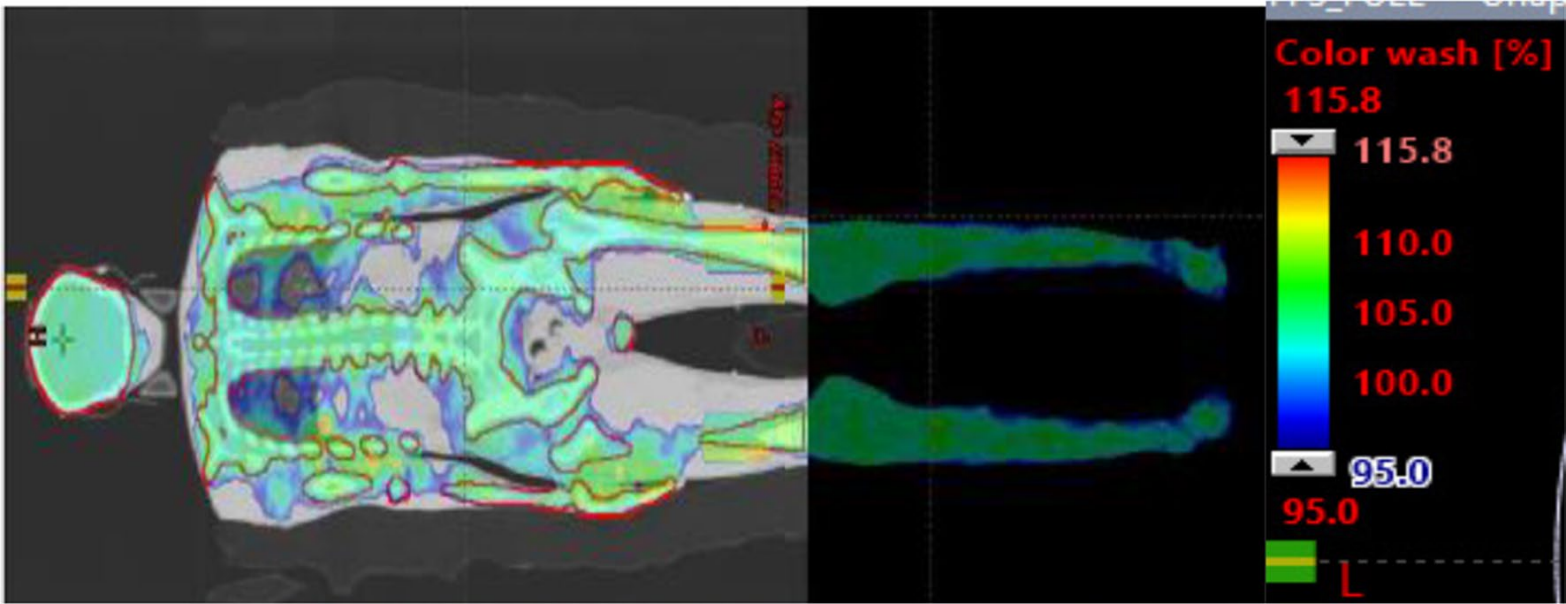
Summed dose distribution: HFS Plan + FFS Plan + HFS junction plan

**Fig. 6:**
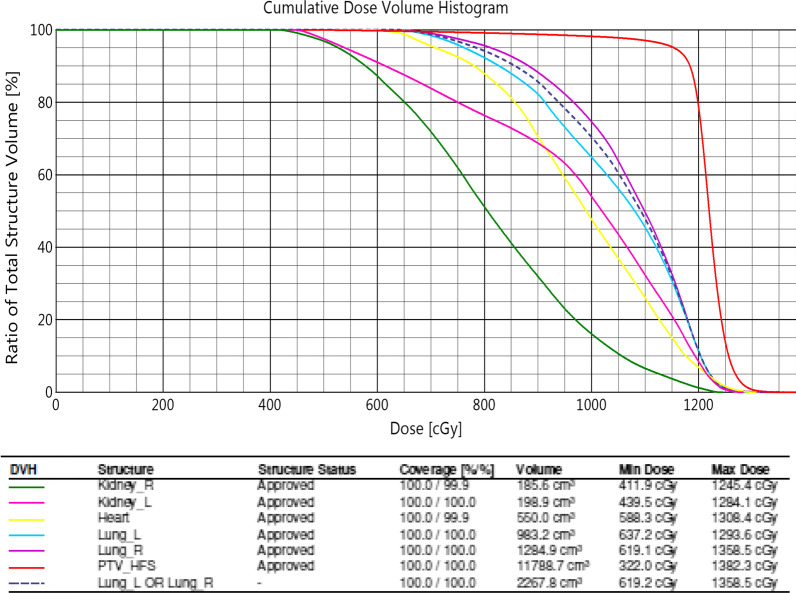
Dose-volume histogram for the patient in Fig. 3

Four patients were simulated, planned, and patient-specific quality assurance, and a dry run was carried out for our center's TMI/TMLI treatment intent. Figure 4 present the dose distribution of four patients in two different axial position. Only one of the patients, a 32-year-old woman with a body weight of 62 kg, a height of 163 cm, and diagnosed as AML, received treatment. Others dropped out due to the comorbidities and clinical complications they developed between simulation and treatment (≈ 4–5 days). The weight and height of the three untreated patients were 116 kg, 52 kg, and 64 kg, and the height varies between 165 and 176 cm and the age of 35 years, 30 years, and 28 years. Figure 5 shows the summed dose distribution for the HFS + FFS + HFS junction plan. Table 1 presents the mean dose-volume parameters for target volume and organs at risk for all four patients. Maximum (± standard deviation) doses for serial organs and average (± standard deviation) doses for parallel organs were reported. Figure 6 presents the dose-volume histogram for the patient presented in Figs. 3 and 5.

**Table 1:** Mean ± standard deviation of the dose to target volume and organ at risk averaged over four patients

	PTV_D95%	PTV_D90%	PTV_V107%	PTV_DMAX	Mean MU	IMRT F (MU/dose)
Target volume	95.0 ± 0% dose (1140 ± 0 cGy)	97.5 ± 0.7% dose (1177 ± 8.5 cGy)	3.3 ± 3.1%	120.7 ± 7.8% (1448.3 ± 93.3 cGy)	7738.25 ± 1056.6	2.28 ± 0.3

	Organ	Volume (cm^3^)	Dose (cGy)		Organ	Volume (cm^3^)	Dose (cGy)
*Organ at risk dose*							
Maximum dose	Lens L	0.15 ± 0.07	489.5 ± 35.5	Average dose	Lung Rt	1216.95 ± 96.1	1021.5 ± 67.6
	Lens R	0.15 ± 0.07	497 ± 69.2		Lung Rt_V12Gy		15.3 ± 6.2%
	Cochlea R	0.3 ± 0.03	1274.6 ± 132.1		Lung Rt_V5Gy		97.9 ± 3.0%
	Cochlea L	0.3 ± 0.04	1271.2 ± 120.6		Whole lung (mean)	2103.3 ± 232.6	1048.6 ± 107.1
Average dose	Eye Rt	8.9 ± 1.3	708.1 ± 56.3		Whole Lung_V12Gy		19.5 ± 12.1%
	Eye Lt	7.8 ± 1.1	655.3 ± 89.3		Whole Lung_V5Gy		98.9 ± 1.6%
	Thyroid	18.3 ± 2.7	1238.3 ± 128.7		Heart	789.8 ± 339.0	921.75 ± 89.2
	Larynx	4.9 ± 1.6	805.1 ± 119.7		Liver	2990.6 ± 1300	820.55 ± 49.7
	Esophagus	32.9 ± 14.5	1197.3 ± 93.1		Stomach	501.5 ± 63.5	1058.3 ± 53.2
	Parotid Left	33.3 ± 6.9	799.3 ± 145.2		Bowel bag	5413.7 ± 890.6	897.9 ± 89.3
	Parotid Right	34.7 ± 7.3	806 ± 131.6		Kidney Rt	214.2 ± 40.4	805.9 ± 9.7
	Oral Cavity	71.7 ± 37.8	811 ± 52.3		Kidney Lt	221.95 ± 32.6	917.9 ± 63.2
	Lung Lt (Mean)	886.8 ± 136.3	1100.05 ± 78.7		Rectum	65.6 ± 17.6	1158.3 ± 230.0
	Lung Lt_V12Gy		25.5 ± 20.6%		Bladder	189.7 ± 49.6	1252.8 ± 105.6
	Lung Lt_V5Gy		100 ± 0%		Ovary	8.4	1238.4
					Scrotum	82 ± 25.3	1220.5 ± 226.7

*D95% and D90%* Percentage dose receiving 95% and 90% volume, *V107%* percentage volume receiving 107% prescription dose, *Dmax* Maximum dose

Mean dose coverage normalized to 95% of the prescription dose received by 95% of the target volume (D95%) is 95.0 ± 0% Dose (1140 ± 0 cGy). The mean 90% dose coverage was 97.5 ± 0.7%. The average Monitor Unit was 7738.25 ± 1056.6. The median number of isocenters for sum of HFS and FFS plans were 17, MU/Dose (cGy) ratio per isocenter was 2.28 ± 0.3. A total of 24 organs were evaluated for the dose-volume parameters as presented in the Table 1. Average organ volume, maximum dose for serial organs, mean dose for parallel organs, and volume receiving 12 Gy (V12Gy), 5 Gy (V5Gy) for combined and bilateral lungs averaged four planned patients. Most critical was lung dose, and the whole lung mean dose is 1048.6 ± 107.1 cGy, and almost full lung is getting a 5 Gy dose bath. The overall mean dose to all organs, excluding serial structures, was found to be 956.1 ± 181.2 cGy. If organs were divided into small (< 100 cm^3^) and large (> 100 cm^3^) categories, overall mean doses for both the groups were 941.7 ± 235.5 cGy and 966.3 ± 139.4 cGy, respectively, indicating similar doses irrespective of the size.

### Patient-specific quality assurance

**Fig. 7:**
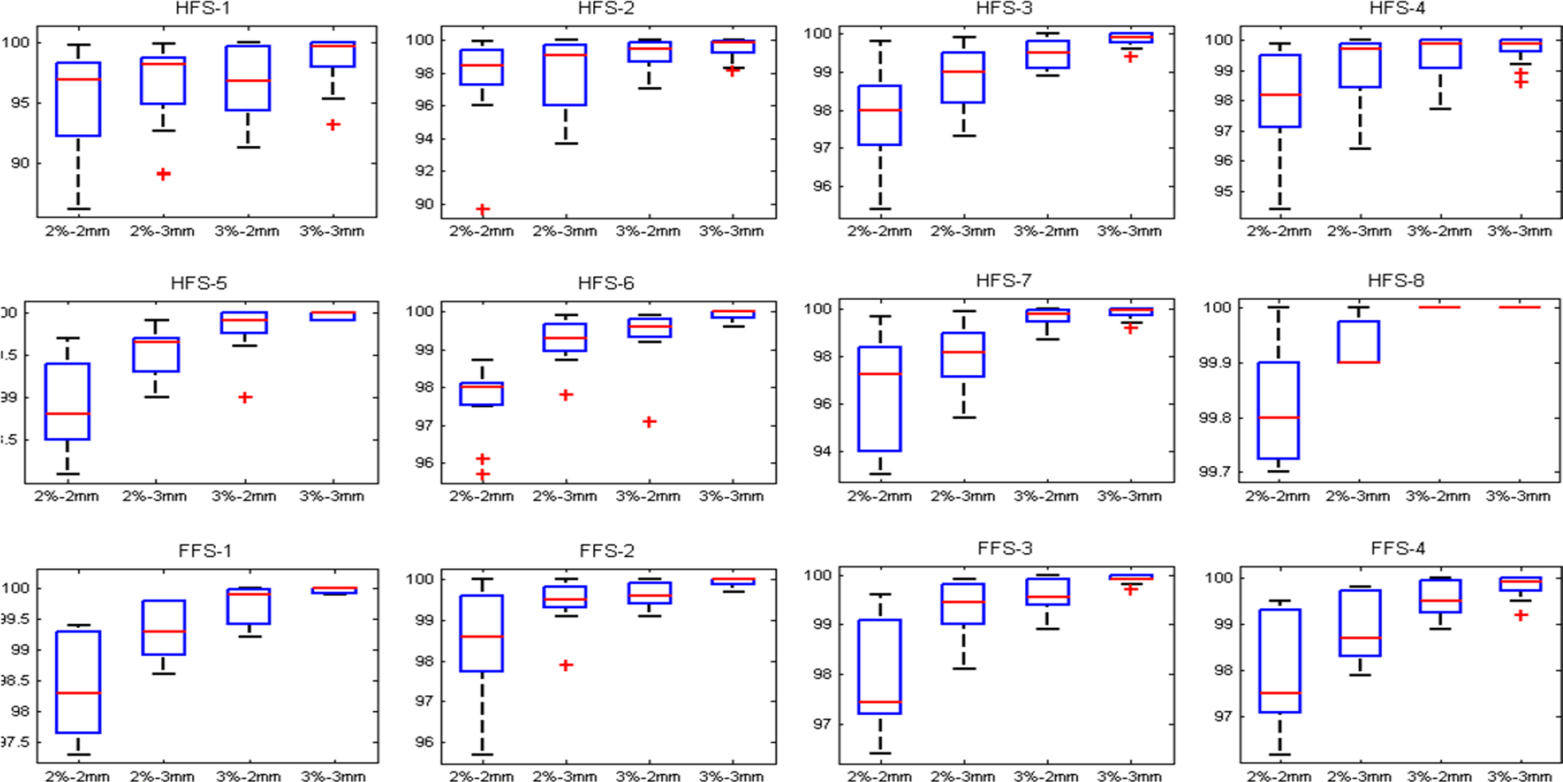
Left panel: Shows the gamma histogram analysis 2%(DD)–2 mm (DTA), 2%–3 mm, 3%–2 mm and 3%–3 mm, averaged over all four patients per isocenter basis

**Fig. 8:**
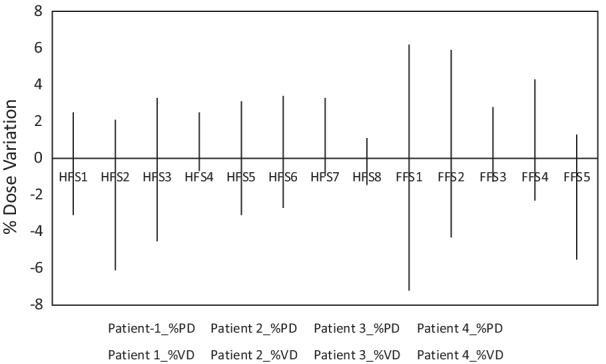
Dose to MU verification for four patients per isocenter PD = point dose and VD is volumetric dose

Dose to MU verification was done by merging all arcs in each isocenter and shows an overall agreement of <− 6.1|0.1 ± 3.0|6.2>% (<lower limit| mean ± standard deviation| upper limit>) for point dose variation and <− 3.1|0.4 ± 2.0|3.9>% for volumetric dose variation. EPID dosimetry 2%–2 mm, and 3%–3 mm gamma passing rates were <93|97.8 ± 2.4|100>%, and <93.2|99.7 ± 0.8|100>% respectively. Figure 7 shows the histogram analysis of gamma passing as a function of different isocenters for various %DD-DTA agreements. Figure 8 presents the dose to MU verification result as a function of isocenters. Optimization time is depends on the body weight and length and varies between 1 and 3 h depending upon the complexity of the dose distribution and the number of runs required to achieve a clinically acceptable dose distribution.

TMLI was followed by allogeneic BMT 3 days after completion of the radiotherapy. The average couch time varies between 83 and 91 min. Translational shifts averaged over all isocenters in lateral, longitudinal, and vertical directions were − 0.4 ± 0.5 cm, − 0.1 ± 0.2 cm, and 0.3 ± 0.3 cm, respectively.

The patient who was able to successfully complete the TMLI and BMT procedures was doped with the marrow of her 23-year-old brother. The HLA typing patient and donor have a 05/10 allele match when tested on peripheral blood in ethylenediaminetetraacetic acid (% match at each locus). After the BMT/TMLI procedure, the patient developed grade 3 gastrointestinal (GI) toxicity and skin erythema. GI toxicity is reflected as diarrhea. In allogeneic transplants, post-BMT complications were caused by graft versus host bone marrow reaction, with little or no contribution from radiotherapy, according to the hemato-oncology team. This is due to the fact that the BMT team had previously encountered similar side effects while using only a chemotherapy-based conditioning regimen without TMLI. The patient was discharged from the hospital after the post-therapy complications were resolved. A successful bone marrow transplant was achieved with the first halcyon-based TMLI.

## Discussion

This study describes the TMI/TMLI treatment, treatment planning, and preclinical validation using the Halcyon-E ring gantry linear accelerator for the first time. We were only able to treat one patient; the others had to be dropped due to clinical complications.

The patient cannot be moved from one treatment isocenter to another using the Halcyon couch system; the only allowed movement is between the CT isocenter and any other treatment isocenter. Therefore after completion of the treatment in any isocentre, the patient must be moved to the CT isocenter where the machine will automatically recognize the next isocenter and shift can be applied. It takes a lot of time to move the patient to and fro each location 15 times on average. The increased number of isocenters will increase to and fro movement between the CT and treatment isocenters.

The isocenter placement strategy is described in both the material and methods section and the Additional file 1. The following strategy needs to be used to decide how to optimize (minimize) the number of arcs per isocenter. The acceptability of the dose distribution is heavily influenced by the arc number per isocenter. According to the current study brain and neck region requires at least two arcs. But for effective dose modulation, it was necessary to have three to four arcs per isocenter from the thorax to the pelvis. The excess of arcs creates two-step problems. First it adds to the optimizer's workload and lengthens the optimization process. Secondly, it will increase the treatment time without any effective improvement in the dose distribution.

In recent time Uehara et al. from Kindai University, Osaka, Japan described the Halcyon based TBI planning [16]. Previously, several authors have described TMI and TBI techniques using the C-ARM Linear Accelerator and ring gantry linear accelerator—tomotherapy [7, 9, 10, 17–20]. Nonetheless, the most common TBI technique is the extended SSD method where the patient is lying inside a box. The gap between the patient and the box is filled with tissue equivalent fillers (like rice) [21, 22]. Treatment was done using a single lateral field, keeping the accelerator gantry at 90°/270° position with the patient placed at an extended distance. Although this process is very complex, labor-intensive, and time-consuming, it has no dependency on (1) patient positional inaccuracy, and (2) open field treatment delivery is certain and without any machine parameter dependency.

Our group has sufficient experience with this box TBI technique, and it takes no less than 1.5 h for a single therapy session [23]. Additionally, it is very inconvenient for patients, and the number of people involved is high, increasing the risk of infection for a highly immunocompromised patient. Patients with aesthesia introduce another layer to the hurdle. The older techniques, such as the one in the standing position, take around 40 min to deliver, which is a very inconvenient technique for patients, hence not in use anymore in clinical practice [17]. An easy solution could be the Halcyon or Tomotherapy based TMLI [7, 16, 24–28]. These techniques reduce the OAR doses significantly. Hui et al. showed OAR dose reduction by 34–70% between TBI and TMI plans [7]. Nonetheless, Tomtherapy based TMLI/TMI is only limited to a very few centers [7–9, 18–21] and has not yet replaced the TBI on a large scale [21, 29]. This has several reasons. First of all, the availability of Tomotherapy is minimal compared to C-ARM linacs in clinics [28]. Use of MV volumetric setup imaging and sliced delivery leads to inefficient setup verification and longer therapy delivery (> 2+ h for an adult patient). Halcyon provides an acceptable dose distribution, faster volumetric delivery, automatic isocenter selection, on-board kV volumetric image acquisition, and artificial intelligence based image matching. The only limitation in all kinds of available VMAT-based techniques (Tomotherapy, Halcyon and C-arm linacs) is the limited table movement which necessitates toggling between head first and feet first treatment except for pediatric patients.

Several studies can be found on TMI/TMLI dose and its comparison between the techniques like IMRT or VMAT and comparative analysys between C-ARM and Tomotherapy linear accelerators like. Mancosu et al. identified total of 12 articles published between 2012 and 2018 feasibility planning studies, optimization of the plan parameters, plan robustness, and dosimetric plan verification [30]. A complete list of the planning studies can be found in Mancosu et al. references [20–31]; however, we restrict our discussion to the major studies temporally spread over the period of observation (2015–2022), as the dosimetric result for the rest of the studies will be a subset of the quoted articles. Among C-ARM based techniques, Foglieta [9], Aydogan [10] and Litoborska [31] published comparative dosimetric analysys for 12 Gy 6 fractions. Mean/median dose Lung 5.5–7.2 Gy, heart 5.2–5.7 Gy, Kidneys ≈ 4.6–5.5 Gy, Liver 5.6–6.3 Gy, Bowel 5.5–7.3 Gy, Brain 5.2–7.4 Gy, Eyes 4.5–6.0 Gy, Lens 3.0–4.0 Gy and oral cavity 2.3–6.1 Gy. Similarly, four temporally separated studies in tomotherapy were Hui et al. [7], Schultheiss [25], Nalichowski et al. [26], and Haraldsson [27]. Mean dose for lung varies between 5.1 and 9.2 Gy, heart between 2.9 and 7.3 Gy, Liver 4.0–8.2 Gy, small bowel 4.5–7.3 Gy, Kidney between 3.5 and 7.4 Gy, eys 3.9–5.4 Gy. The doses reported in C-Arm based TMI/TMLI studies are nearly uniform whereas tomotherapy studies show a significant dose difference. Nalichowski et al. reported doses were much lesser than in other studies [26].

Doses reported in the present study are higher than the other reported studies in Tomotherapy or C-ARM linac. Probably due to the fact that we are at the early part of the learning curve. It requires incubation time to standardise the contouring and planning. This study, with the involvement of actual patients, was not a typical planning study where contouring and planning were done with no time limit; hence, results may not be as refined as in a planning study. We are hopeful that with a few more cases we will be able to reduce the OAR doses. Additionally, patients we received were obese in nature. We observed the dose distribution was highly susceptible to the height and weight of the patients. Our first patient was a 170 cm tall female patient with a body weight of 116 kg. It was challenging to achieve the pulmonary dose, but other dose constraints were met. For lean patients with less body weight, it is much easier to achieve the OAR doses, including pulmonary doses (Mean and V12Gy).

Although the total dose is less, the full dose to all the organs is attributed to increased late toxicities due to radiation, especially for pediatric patients [5]. This includes neurocognitive decline, growth impairment, long-term endocrinological toxicity, and secondary malignancies [5, 6]. For adult patients, late toxicity may manifest as a risk of lung damage and might lead to a significant clinical concern [5, 6]. Several authors have presented the different OAR toxicities attributed to TBI dose delivery, references [3–24] in the Hui et al. report [7]. Late lung toxicities and cataracts are more prevalent among children [32–42]. Some investigators have suggested lung shielding using hands to reduce the pulmonary dose, which is an effective way and offers a natural reproducible position between different therapy sessions [36]. Similarly, different renal [36, 37], cardiac [38–40] and liver (veno-occlusive disease) [41, 42] complications were contributed from chemo-radiotherapy or radiotherapy alone. Because of the long life expectancy attributed to the high cure rate of BMT patients, it is essential to preserve pulmonary function. Nonetheless, these OAR doses are deduced from TLD, diode, film, or MOSFET measurements placed on the patient's surface. Dose calculations are based only on phantom measurements without tissue heterogeneity corrections for lung or bone [26, Q: 24]. These measurements have some degree of uncertainty. Our TMI planning dose calculation based on CT data set in a well-established dose calculation engine offers better accuracy. In the present study, the planning strategy includes a set of pre-decided dose contaminants from the literature. We could achieve all of them except the lung V12Gy dose for one obese patient. We are at the beginning of the learning curve with only three patients planned. We expect to improve the dose distribution with subsequent patients. Several authors have compared the Tomotherapy.

## Conclusion

The present study demonstrates the treatment along with the planning and pre-clinical validation of total marrow irradiation and total marrow and lymph-node irradiation using the Halcyon-E ring gantry linear accelerator for the first time. It produces a clinically acceptable plan and much faster delivery than conventional TBI Box techniques, reducing patient contact with the staff and lowering the risk of infection for highly immune-compromised patients. PTV dose coverage and OAR dose constraints to OARs were achievable except for one or two exceptions, like lung V12Gy for obese patients.

## Supplementary Information

**Additional file 1.** Radiotherapy planning detail of TMLI. Arc and isocentre placement strategy, optimization parameters, and dose distribution.

## Data Availability

Available with corresponding author. The datasets used and analysed during the current study are available from the corresponding author.

## References

[1] Copelan EA (2006). Hematopoietic stem-cell transplantation. N Engl J Med.

[2] Brochstein J, Kernan N, Groshen S (1987). Allogeneic bone marrow transplantation after hyperfractionated total-body irradiation and cyclophosphamide in children with acute leukemia. N Engl J Med.

[3] Thomas E, Storb R, Clift RA, Fefer A, Johnson FL, Neiman PE, Lerner KG, Glucksberg H, Buckner CD (1975). Bone-marrow transplantation (first of two parts). N Engl J Med.

[4] Thomas ED, Storb R, Clift RA, Fefer A, Johnson FL, Neiman PE, Lerner KG, Glucksberg H, Buckner CD (1975). Bone-marrow transplantation: (second of two parts). N Engl J Med.

[5] Cosset JM, Socie G, Dubray B, Girinsky T, Fourquet A, Gluckman E (1994). Single dose versus fractionated total body irradiation before bone marrow transplantation: radiobiological and clinical considerations. Int J Radiat Oncol Biol Phys.

[6] Sanders JE (1990). Late effects in children receiving total body irradiation for bone marrow transplantation. Radiother Oncol.

[7] Hui SK, Kapatoes J, Fowler J, Henderson D, Olivera G, Manon RR, Gerbi B, Mackie TR, Welsh JS (2005). Feasibility study of helical tomotherapy for total body or total marrow irradiation a. Med Phys.

[8] Wilkie JR, Tiryaki H, Smith BD, Roeske JC, Radosevich JA, Aydogan B (2008). Feasibility study for linac-based intensity modulated total marrow irradiation. Med Phys.

[9] Fogliata A, Cozzi L, Clivio A, Ibatici A, Mancosu P, Navarria P, Nicolini G, Santoro A, Vanetti E, Scorsetti M (2011). Preclinical assessment of volumetric modulated arc therapy for total marrow irradiation. Int J Radiat Oncol Biol Phys.

[10] Aydogan B, Yeginer M, Kavak GO, Fan J, Radosevich JA, Gwe-Ya K (2011). Total marrow irradiation with rapidArc volumetric arc therapy. Int J Radiat Oncol Biol Phys.

[11] Wong JY, Filippi AR, Scorsetti M, Hui S, Muren LP, Mancosu P (2020). Total marrow and total lymphoid irradiation in bone marrow transplantation for acute leukaemia. Lancet Oncol.

[12] Sarkar B, Munshi A, Manikandan A, Roy S, Ganesh T, Mohanti BK, Pradhan A (2018). A low gradient junction technique of craniospinal irradiation using volumetric-modulated arc therapy and its advantages over the conventional therapy. Cancer/Radiothérapie.

[13] Sarkar B, Munshi A, Ganesh T, Manikandan A, Mohanti BK (2020). Dosimetric comparison of short and full arc in spinal PTV in volumetric-modulated arc therapy-based craniospinal irradiation. Med Dosim.

[14] Sarkar B, Ghosh B, Sriramprasath SM, Basu A, Goswami J, Ray A (2010). Optimized point dose measurement for monitor unit verification in intensity modulated radiation therapy using 6 MV photons by three different methodologies with different detector-phantom combinations: a comparative study. J Med Phys Assoc Med Phys India.

[15] Mancosu P, Navarria P, Castagna L, Reggiori G, Stravato A, Gaudino A, Sarina B, Tomatis S, Scorsetti M (2015). Plan robustness in field junction region from arcs with different patient orientation in total marrow irradiation with VMAT. Physica Med.

[16] Uehara T, Monzen H, Tamura M, Inada M, Otsuka M, Matsumoto K, Nishimura Y (2021). Feasibility study of volumetric modulated arc therapy with Halcyon™ linac for total body irradiation. Radiat Oncol.

[17] Miralbell R, Rouzaud M, Grob E, Nouet P, Bieri S, Majno SB, Botteron P, Montero M, Precoma JC (1994). Can a total body irradiation technique be fast and reproducible?. Int J Radiat Oncol Biol Phys.

[18] Wong JY, Liu A, Schultheiss T (2006). Targeted total marrow irradiation using three-dimensional image-guided tomographic intensity-modulated radiation therapy: an alternative to standard total body irradiation. Biol Blood Marrow Transplant.

[19] Aydogan B, Mundt AJ, Roeske JC (2006). Linac-based intensity modulated total marrow irradiation (IM-TMI). Technol Cancer Res Treat.

[20] Wilkie JR, Tiryaki H, Smith BD, Roeske JC, Radu CG, Aydogan B (2008). Feasibility study for linac-based intensity modulated total marrow irradiation. Med Phys.

[21] Mesa F, Eng TY, Esquivel C, Fuller CD, Papanikolaou N, Sosa M (2011). Implementation of a lateral total body irradiation technique with 6 MV photons: The University of Texas Health Science Center in San Antonio experience. J Radiother Pract.

[22] Peters M, Taylor B, Turner E (2015). An evidence-based review of total body irradiation. J Med Imaging Radiat Sci.

[23] Karthik V, Osman S, Singh S, Jassal K, Sarkar B, Ganesh T, Giri UK (2017). Utilization of OSLD as the quality control indicator for in-vivo measurements in total body irradiation. J Med Phys.

[24] Sarkar B, Shahid T, Mondal S, Biswal S, De A, Mukherjee M, Roy Chowdhury S, Bhattacharya J, George K, Ghosh T, Soren P, Banik A, Das P, Ghosh S, Gunturu I, Pusarla C, Mishra A, Mitra S. PO-GePV-T-316 Pre-clinical validation and treatment of volumetric modulated arc therapy based total bone marrow irradiation in Halcyon™ ring gantry linear accelerator. Abstract Med Phys. 2022;49:e929. 10.1002/mp.15769.

[25] Schultheiss TE, Wong J, Liu A, Olivera G, Somlo G (2007). Image-guided total marrow and total lymphatic irradiation using helical tomotherapy. Int J Radiat Oncol Biol Phys.

[26] Nalichowski A, Eagle DG, Burmeister J (2016). Dosimetric evaluation of total marrow irradiation using 2 different planning systems. Med Dosim.

[27] Haraldsson A, Engellau J, Lenhoff S, Engelholm S, Bäck S, Engström PE (2019). Implementing safe and robust total marrow irradiation using helical tomotherapy–a practical guide. Physica Med.

[28] Shueng PW, Lin SC, Chong NS, Lee HY, Tien HJ, Wu LJ, Chen CA, Lee JJ, Hsieh CH (2009). Total marrow irradiation with helical tomotherapy for bone marrow transplantation of multiple myeloma: first experience in Asia. Technol Cancer Res Treat.

[29] Wong J, Wong J, Hui SK (2020). Total marrow irradiation: redefining the role of radiotherapy in bone marrow transplantation. Total marrow irradiation.

[30] Mancosu P, Cozzi L, Muren LP (2019). Total marrow irradiation for hematopoietic malignancies using volumetric modulated arc therapy: a review of treatment planning studies. Phys Imaging Radiat Oncol.

[31] Litoborska J, Piotrowski T, Malicki J (2020). Evaluation of three VMAT-TMI planning methods to find an appropriate balance between plan complexity and the resulting dose distribution. Physica Med.

[32] Bruno B, Souillet G, Bertrand Y, Werck-Gallois MC, Satta AS, Bellon G (2004). Effects of allogeneic bone marrow transplantation on pulmonary function in 80 children in a single paediatric centre. Bone Marrow Transplant.

[33] Cogan DG, Donaldson DD, Reese AB (1952). Clinical and pathological characteristics of radiation cataract. AMA Arch Ophthalmol.

[34] van Kempen-Harteveld ML, Belkacémi Y, Kal HB, Labopin M, Frassoni F (2002). Dose-effect relationship for cataract induction after single-dose total body irradiation and bone marrow transplantation for acute leukemia. Int J Radiat Oncol Biol Phys.

[35] Della Volpe A, Ferreri AJ, Annaloro C, Mangili P, Rosso A, Calandrino R, Villa E, Lambertenghi-Deliliers G, Fiorino C (2002). Lethal pulmonary complications significantly correlate with individually assessed mean lung dose in patients with hematologic malignancies treated with total body irradiation. Int J Radiat Oncol Biol Phys.

[36] Miralbell R, Sancho G, Bieri S, Carrió I, Helg C, Brunet S, Martin PY, Sureda A, De Segura GG, Chapuis B, Estorch M (2004). Renal insufficiency in patients with hematologic malignancies undergoing total body irradiation and bone marrow transplantation: a prospective assessment. Int J Radiat Oncol Biol Phys.

[37] Cooper DL, Seropian S, Childs RW (2001). Autologous and allogeneic stem cell transplant. Cancer: principles and practice of oncology.

[38] Murdych T, Weisdorf DJ (2001). Serious cardiac complications during bone marrow transplantation at the University of Minnesota, 1977–1997. Bone Marrow Transplant.

[39] Buja LM, Ferrans VJ, Graw RG (1976). Cardiac pathologic findings in patients treated with bone marrow transplantation. Hum Pathol.

[40] Blum W, Khoury H, Lin HS, Vij R, Goodnough LT, Devine S, DiPersio J, Adkins D (2003). Primary amyloidosis patients with significant organ dysfunction tolerate autologous transplantation after conditioning with single-dose total body irradiation alone: a feasibility study. Biol Blood Marrow Transplant.

[41] Hassan M, Ljungman P, Ringden O, Hassan Z, Öberg G, Nilsson C, Bekassy A, Bielenstein M, Abdel-Rehim M, Georen S, Astner L (2000). The effect of busulphan on the pharmacokinetics of cyclophosphamide and its 4-hydroxy metabolite: time interval influence on therapeutic efficacy and therapy-related toxicity. Bone Marrow Transplant.

[42] Lee JH, Lee KH, Choi SJ, Min YJ, Kim JG, Kim S, Lee JS, Kim SH, Park CJ, Chi HS, Kim WK (2000). Veno-occlusive disease of the liver after allogeneic bone marrow transplantation for severe aplastic anemia. Bone Marrow Transplant.

