# Supplementary material for: Preclinical validation and treatment of volumetric modulated arc therapy based total bone marrow irradiation in Halcyon™ ring gantry linear accelerator
Source: Radiat Oncol. 2022 Aug 19;17:145. doi: 10.1186/s13014-022-02109-z (PMC9389791; doi:10.1186/s13014-022-02109-z)
Supplement: Supplementary file 1 — Additional file 1. Radiotherapy planning detail of TMLI. Arc and isocentre placement strategy, optimization parameters, and dose distribution. [file 13014_2022_2109_MOESM1_ESM.pptx]

## Slide 1
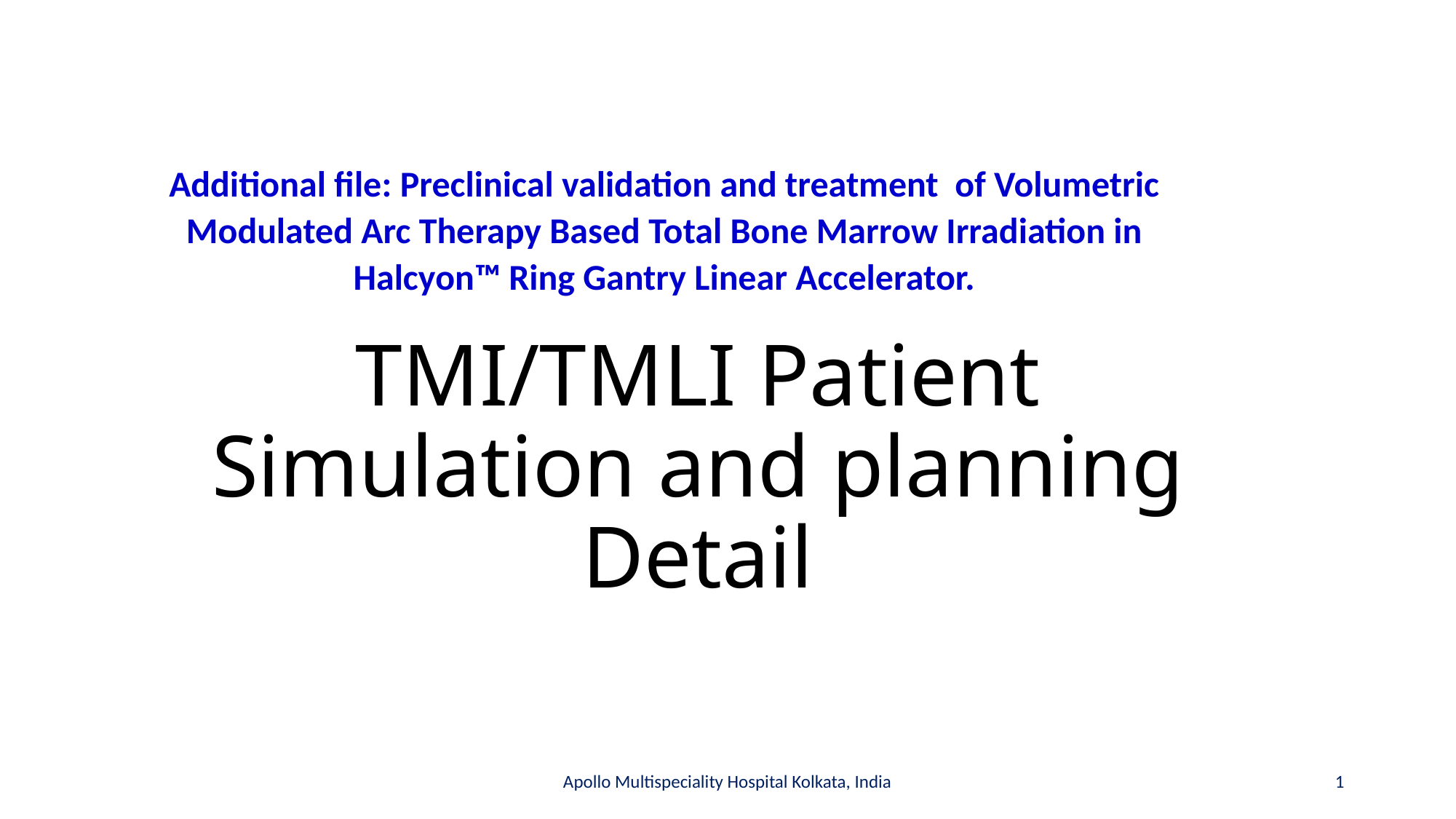

Additional file: Preclinical validation and treatment of Volumetric Modulated Arc Therapy Based Total Bone Marrow Irradiation in Halcyon™ Ring Gantry Linear Accelerator.
# TMI/TMLI Patient Simulation and planning Detail
Apollo Multispeciality Hospital Kolkata, India
1

## Slide 2
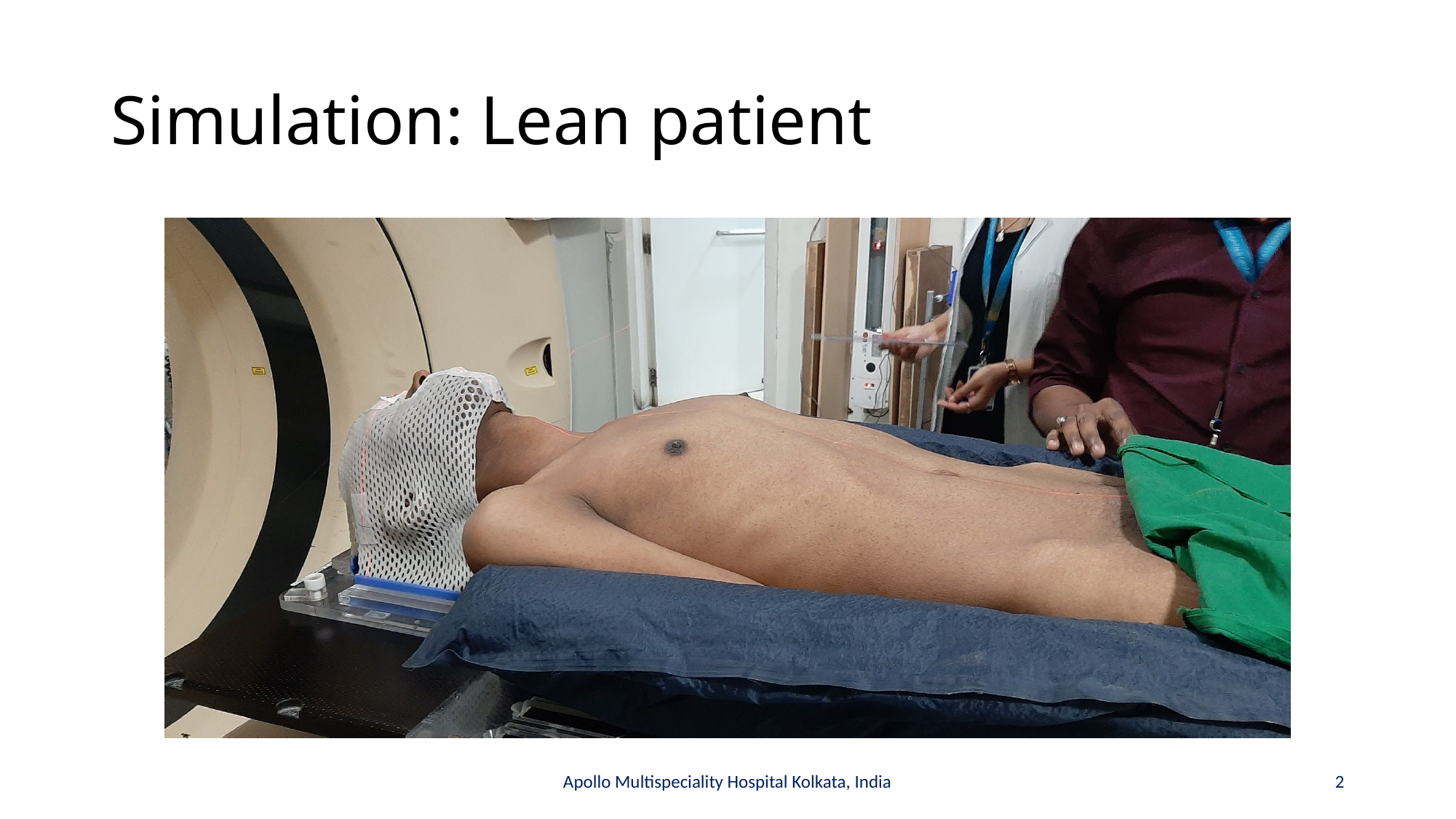

# Simulation: Lean patient
Apollo Multispeciality Hospital Kolkata, India
2

## Slide 3
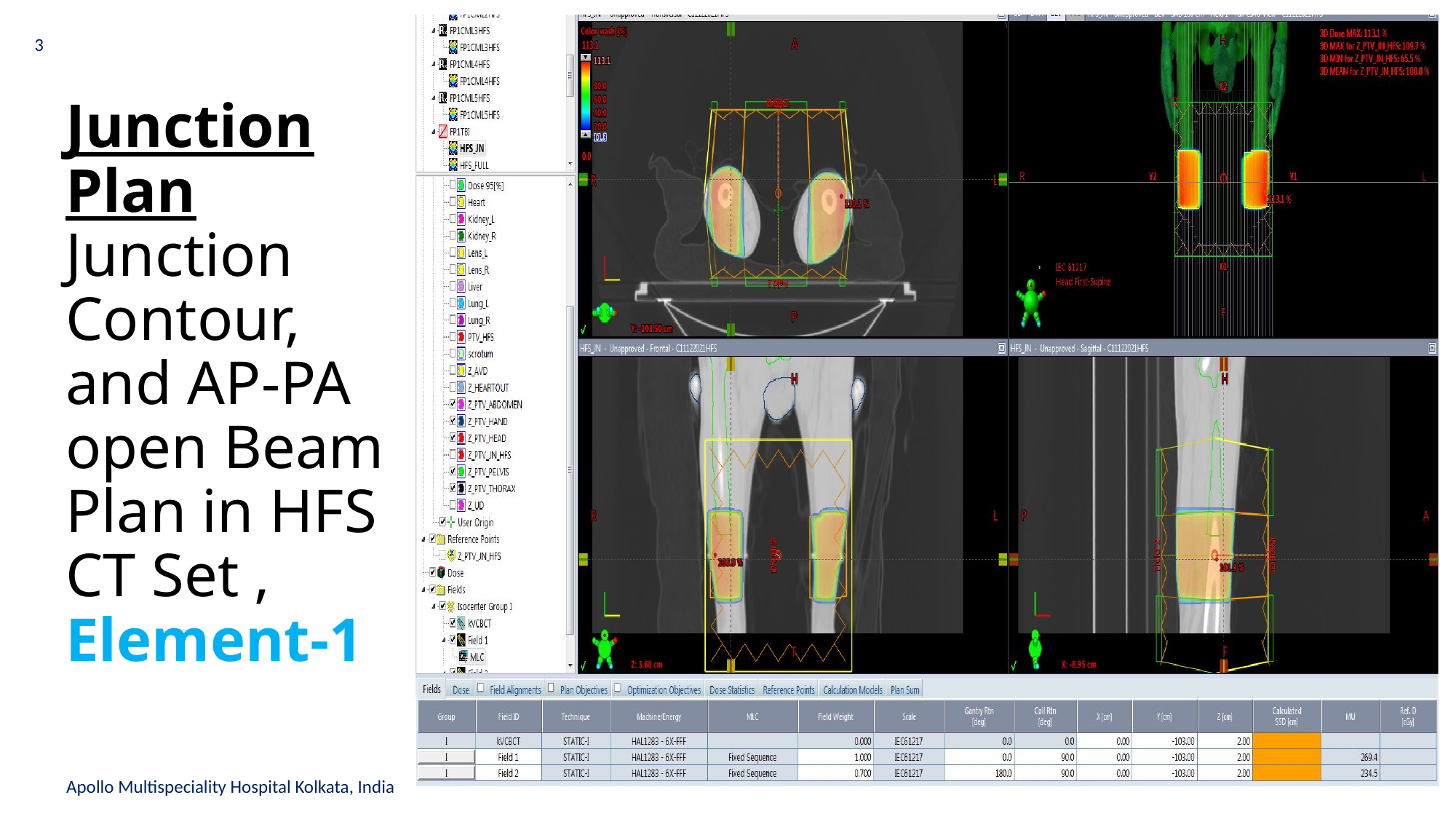

3
# Junction Plan Junction Contour, and AP-PA open Beam Plan in HFS CT Set , Element-1
Apollo Multispeciality Hospital Kolkata, India

## Slide 4
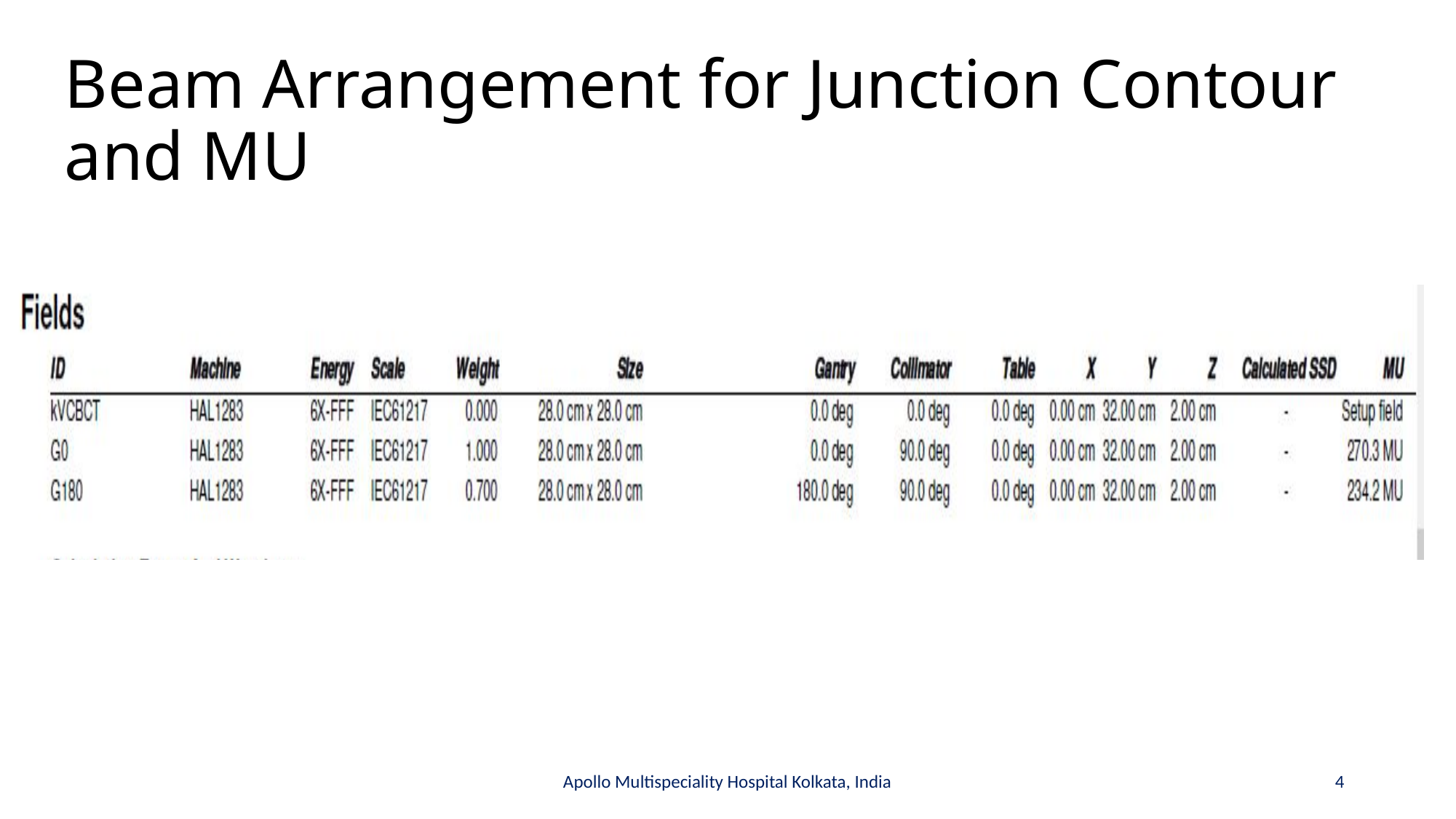

# Beam Arrangement for Junction Contour and MU
Apollo Multispeciality Hospital Kolkata, India
4

## Slide 5
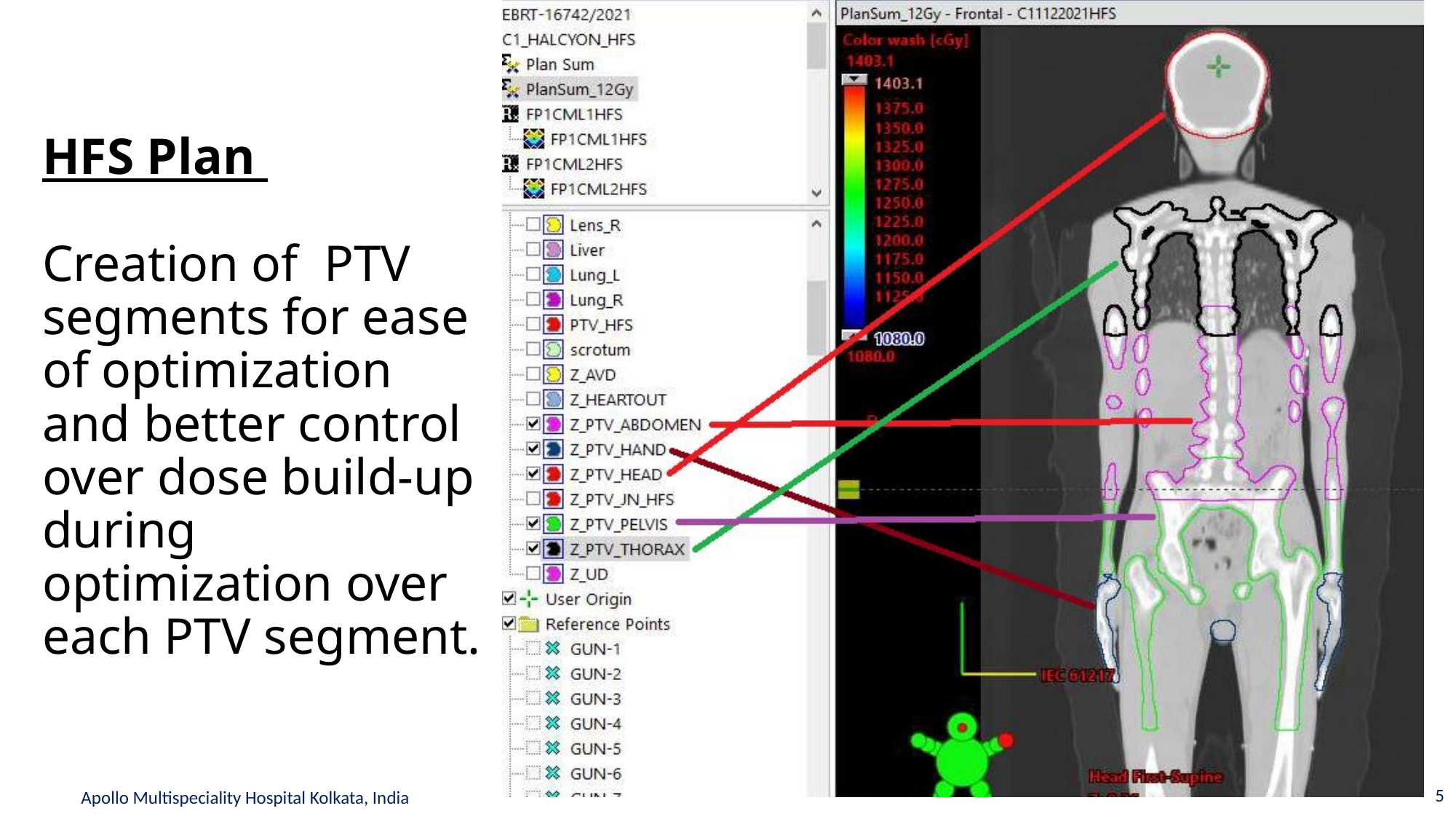

# HFS Plan Creation of PTV segments for ease of optimization and better control over dose build-up during optimization over each PTV segment.
5
Apollo Multispeciality Hospital Kolkata, India

## Slide 6
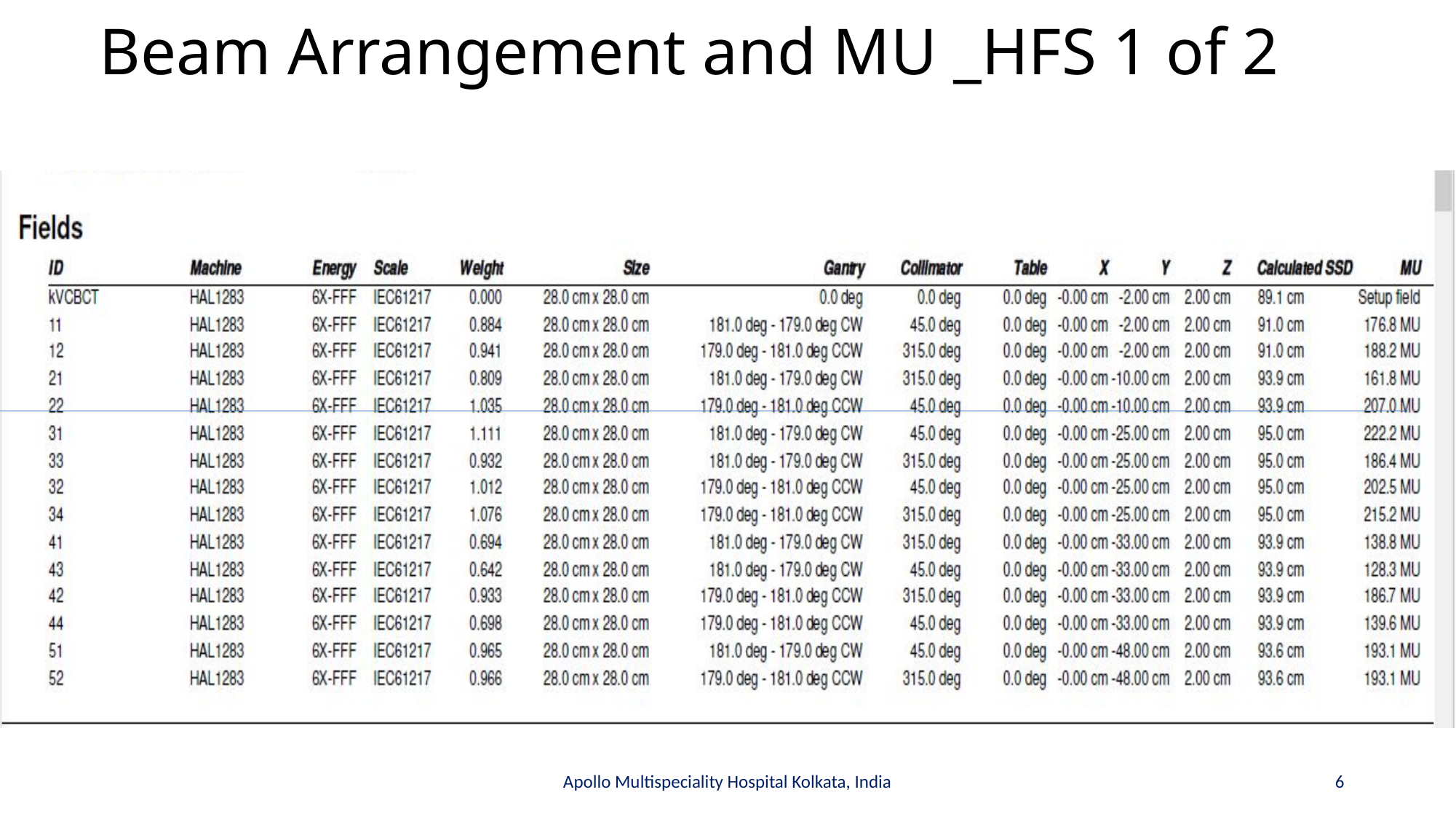

# Beam Arrangement and MU _HFS 1 of 2
Apollo Multispeciality Hospital Kolkata, India
6

## Slide 7
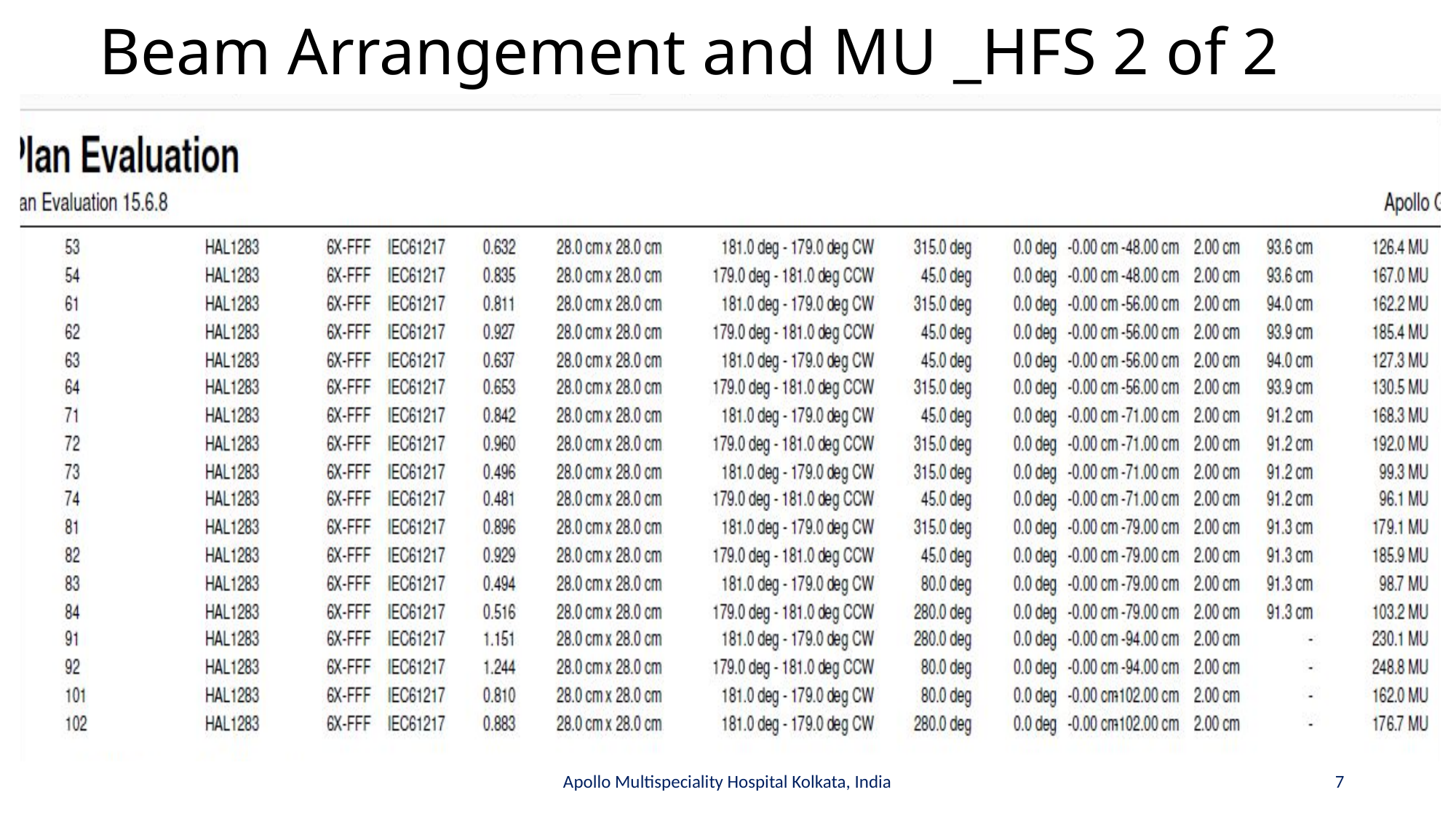

# Beam Arrangement and MU _HFS 2 of 2
Apollo Multispeciality Hospital Kolkata, India
7

## Slide 8
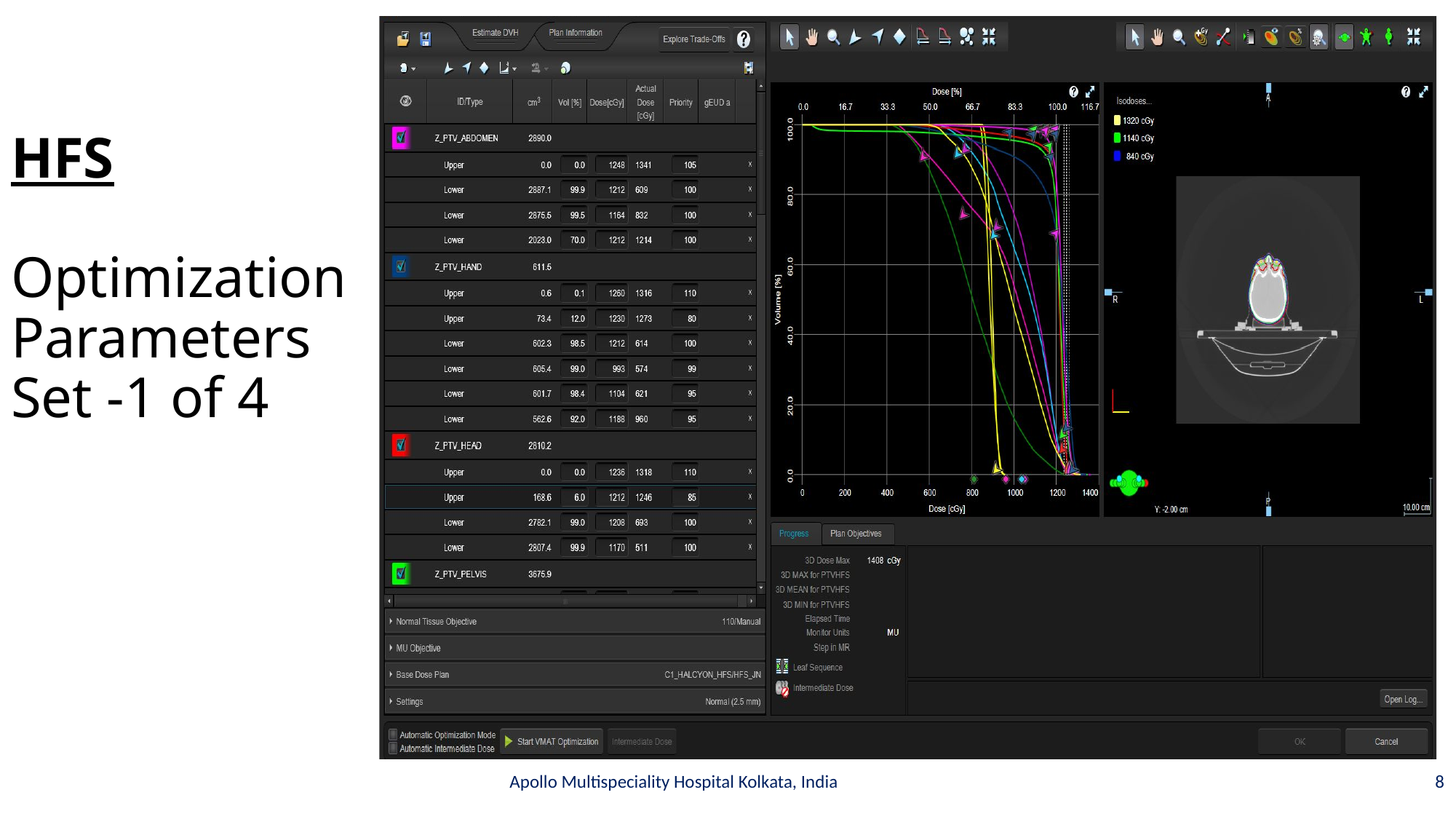

# HFS Optimization Parameters Set -1 of 4
Apollo Multispeciality Hospital Kolkata, India
8

## Slide 9
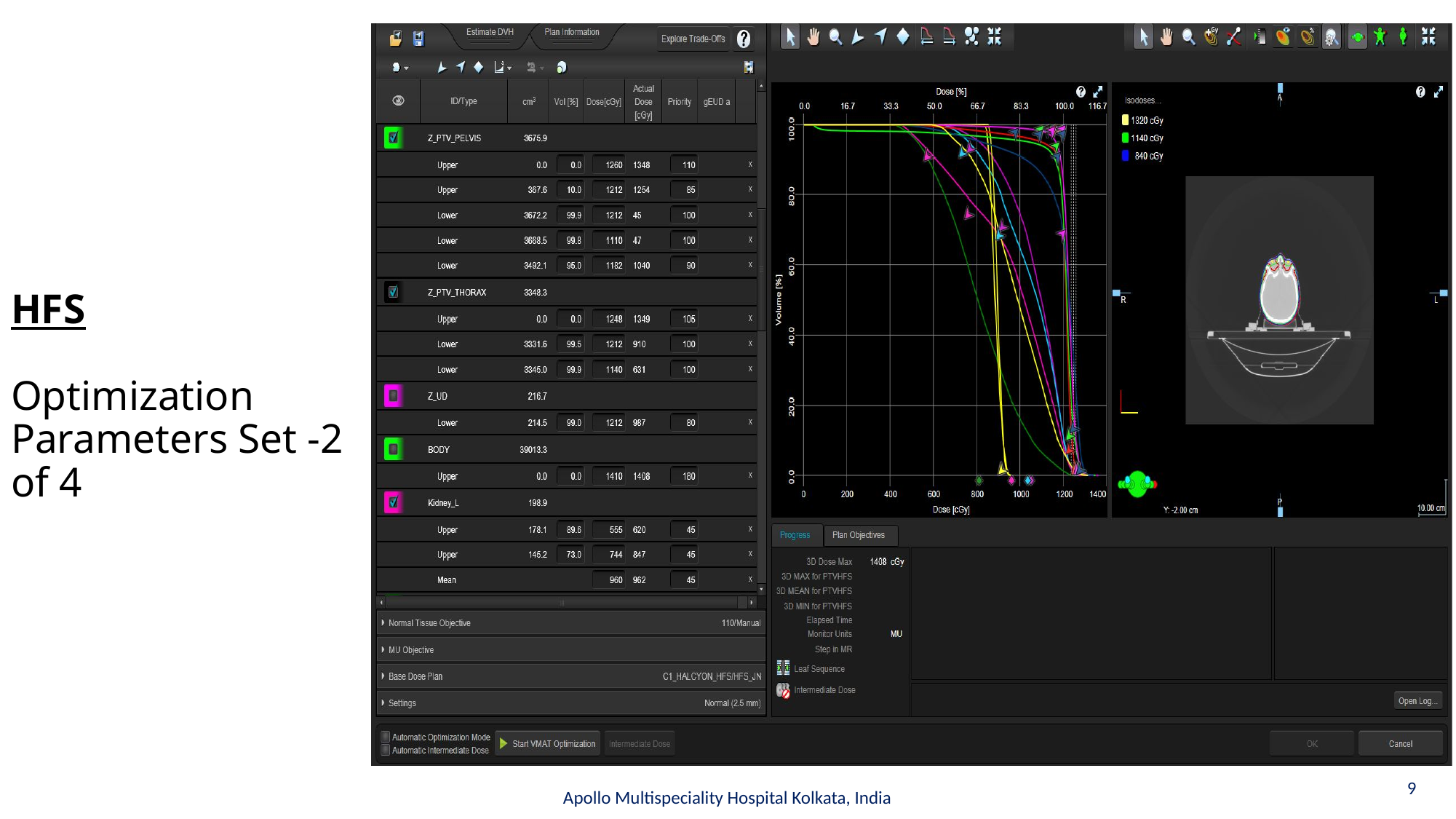

# HFS Optimization Parameters Set -2 of 4
9
Apollo Multispeciality Hospital Kolkata, India

## Slide 10
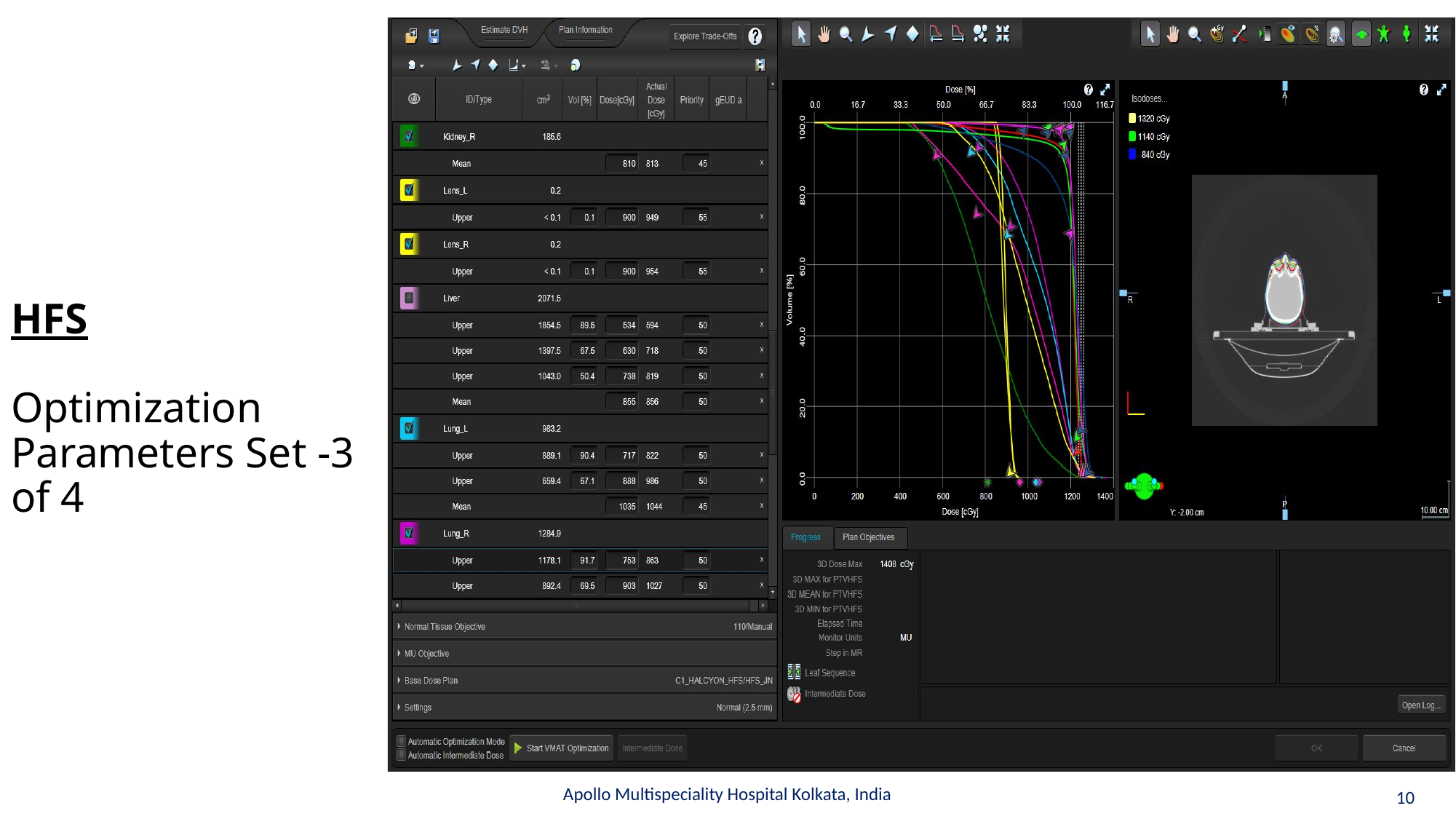

# HFS Optimization Parameters Set -3 of 4
Apollo Multispeciality Hospital Kolkata, India
10

## Slide 11
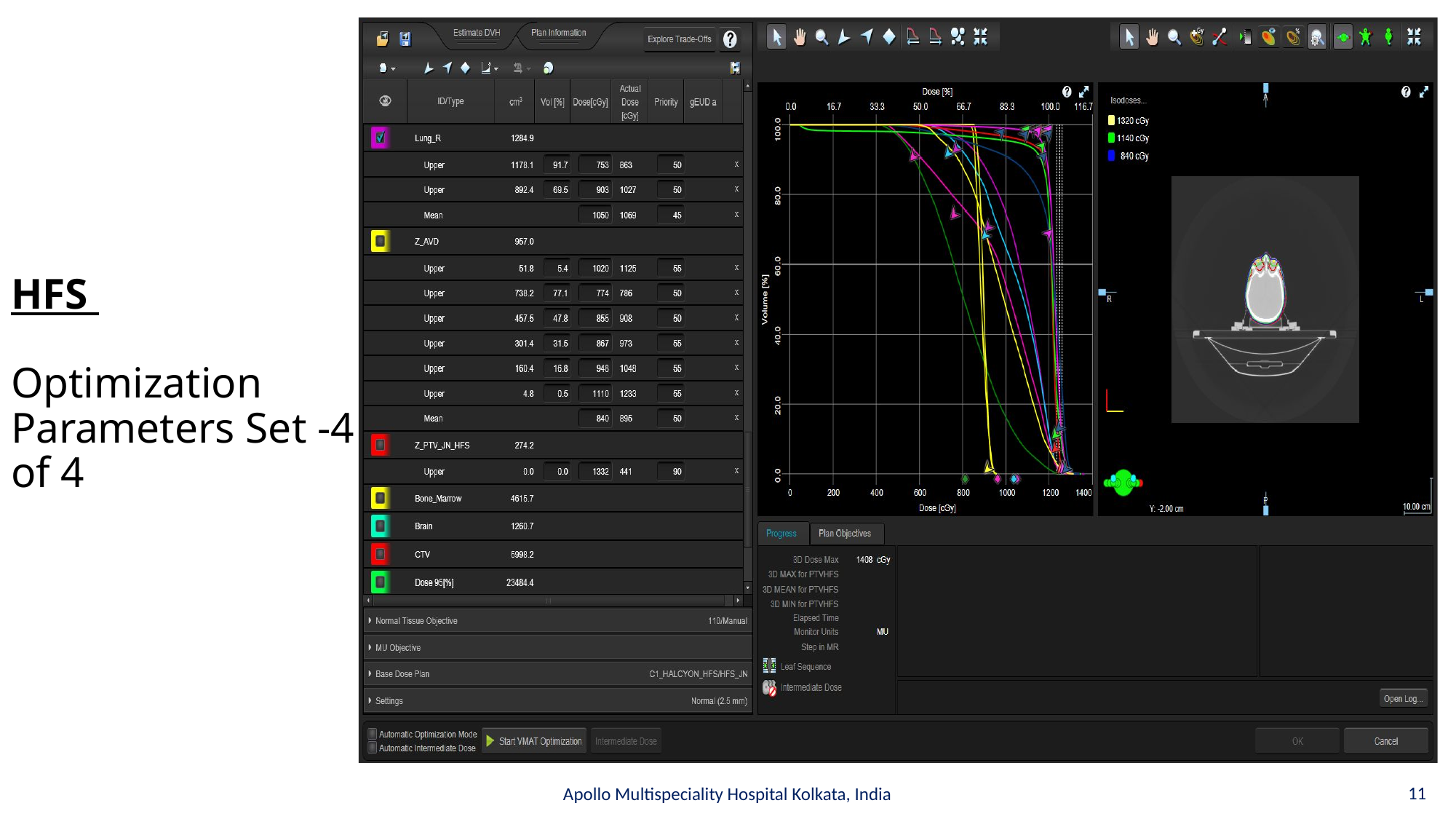

# HFS Optimization Parameters Set -4 of 4
11
Apollo Multispeciality Hospital Kolkata, India

## Slide 12
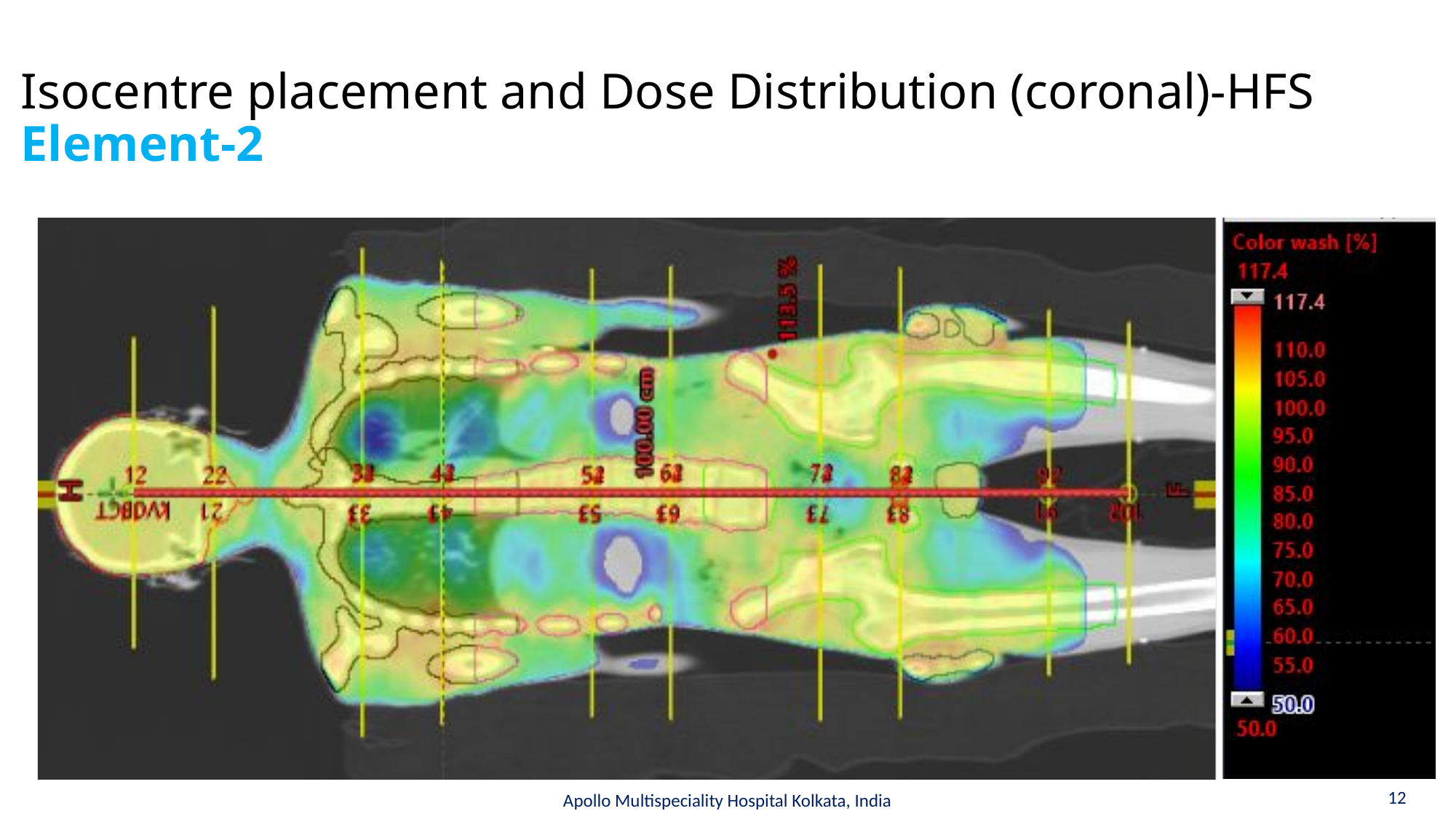

# Isocentre placement and Dose Distribution (coronal)-HFS Element-2
12
Apollo Multispeciality Hospital Kolkata, India

## Slide 13
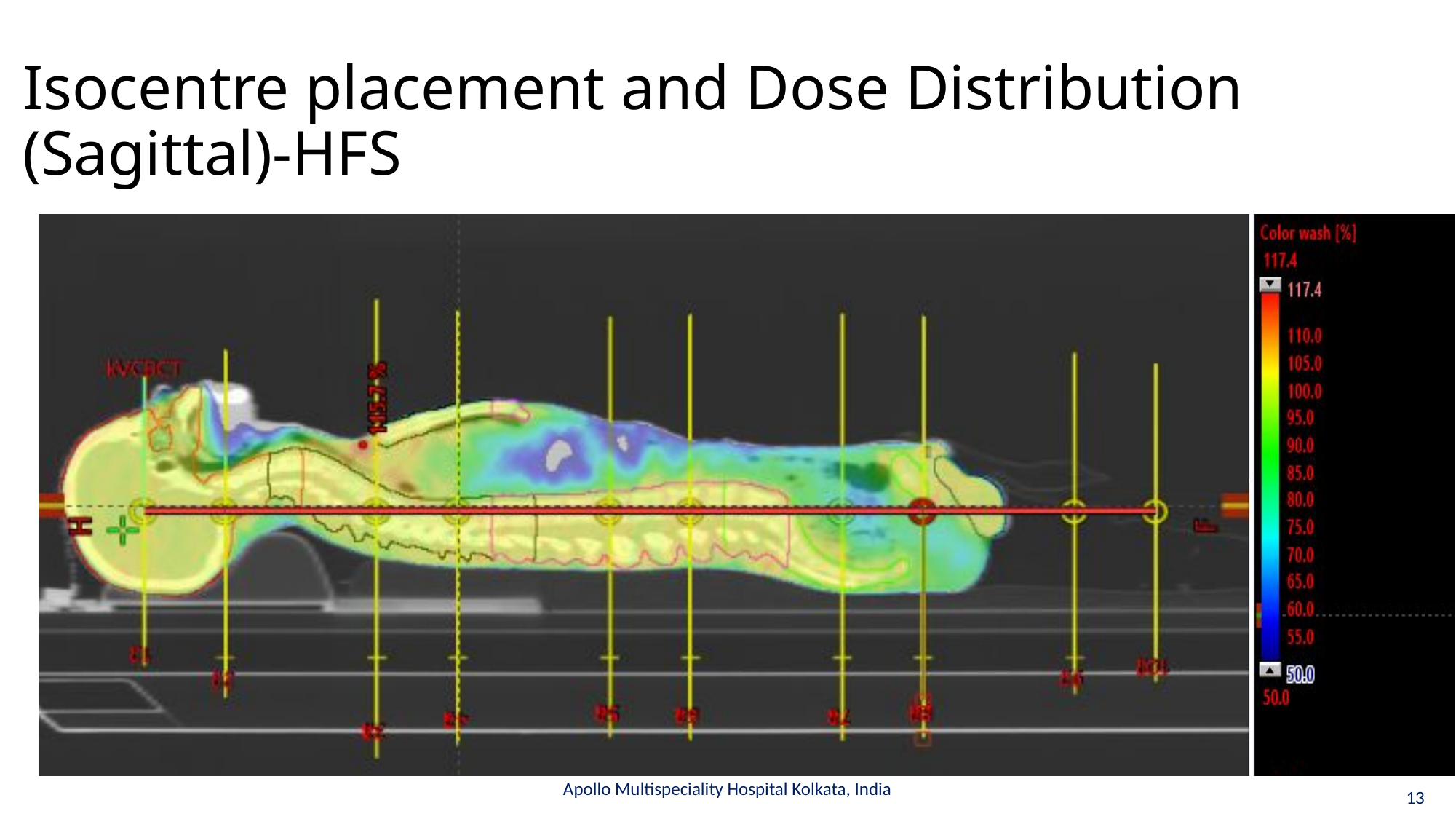

# Isocentre placement and Dose Distribution (Sagittal)-HFS
Apollo Multispeciality Hospital Kolkata, India
13

## Slide 14
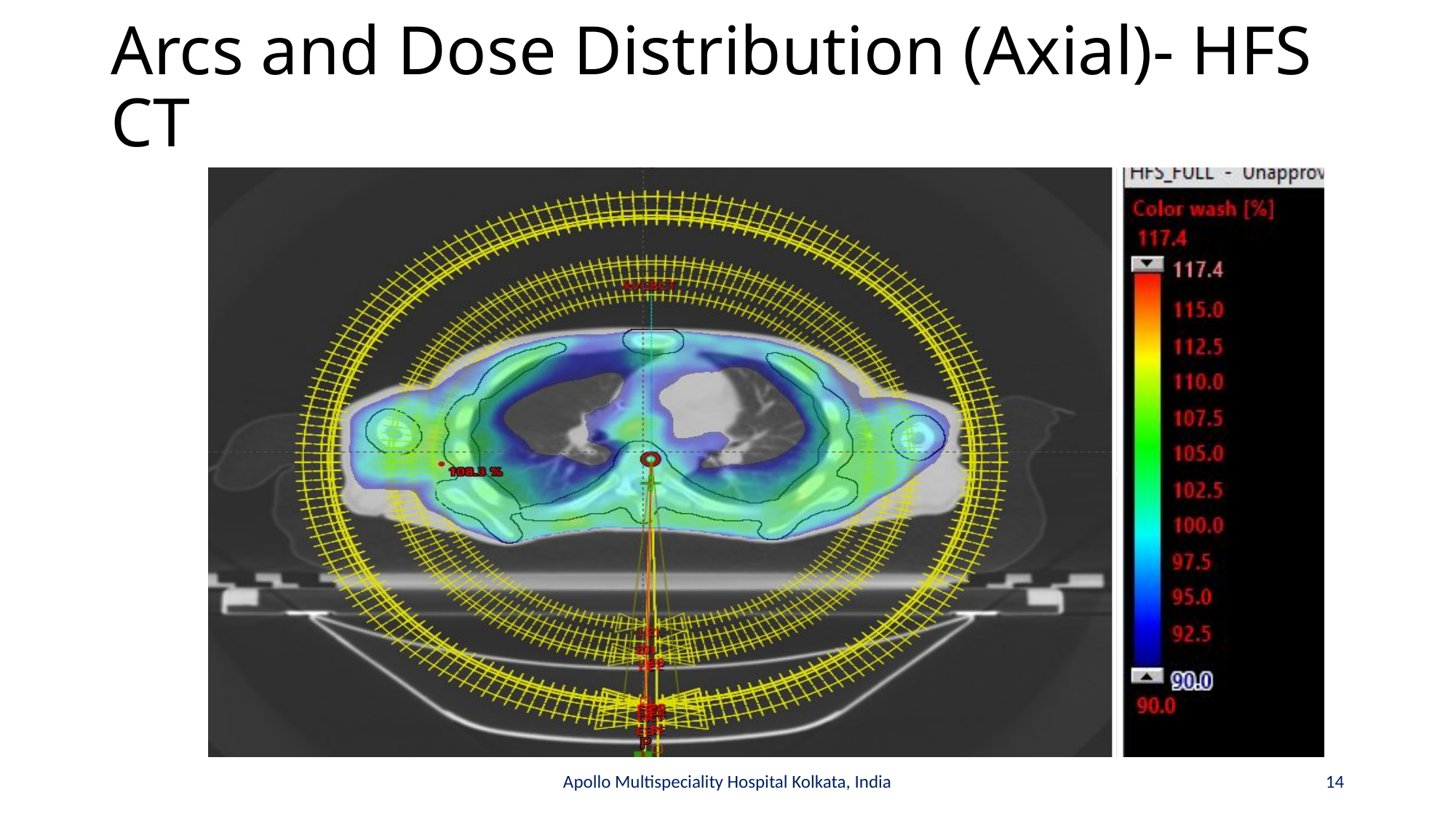

# Arcs and Dose Distribution (Axial)- HFS CT
Apollo Multispeciality Hospital Kolkata, India
14

## Slide 15
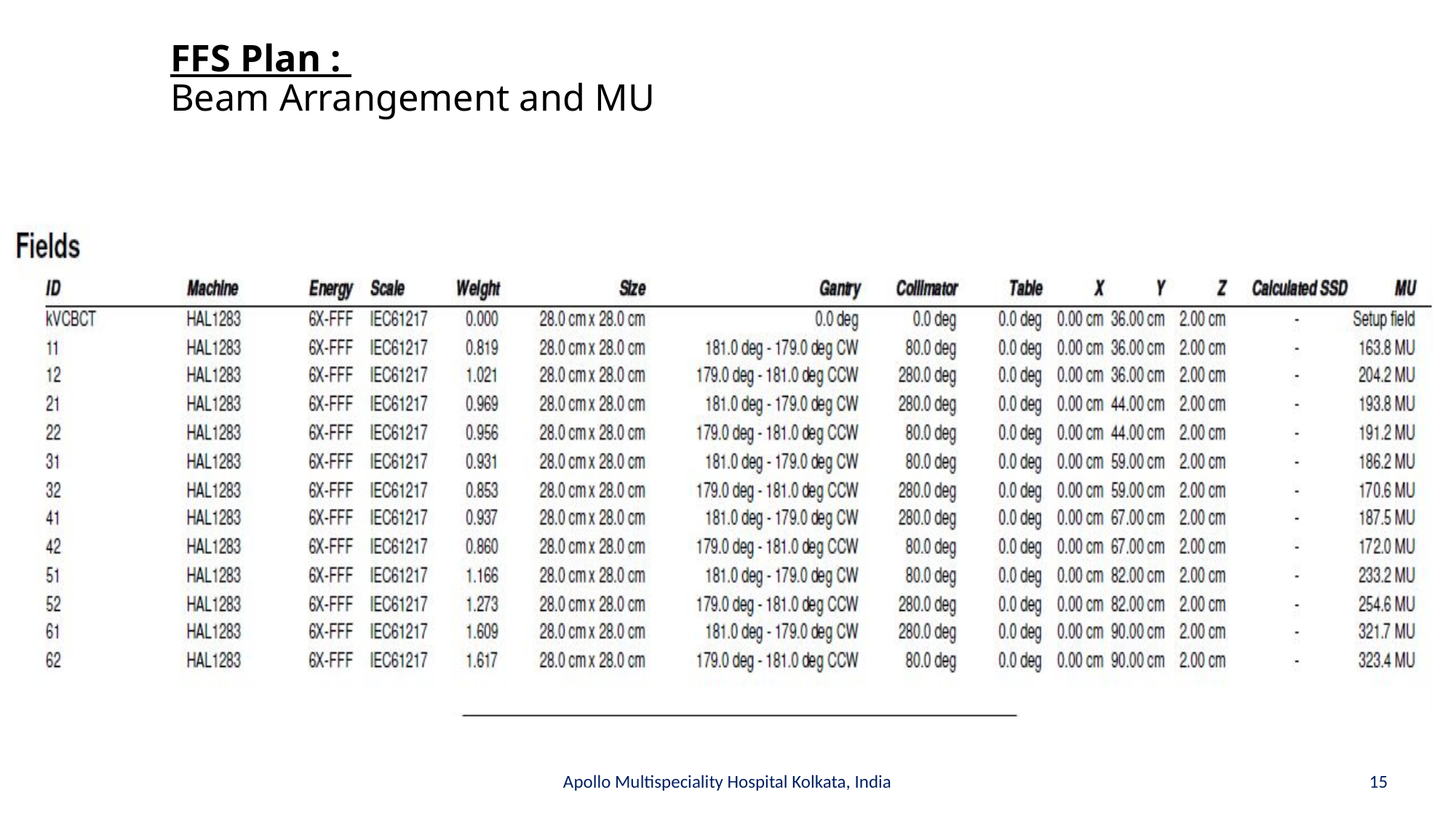

# FFS Plan : Beam Arrangement and MU
15
Apollo Multispeciality Hospital Kolkata, India

## Slide 16
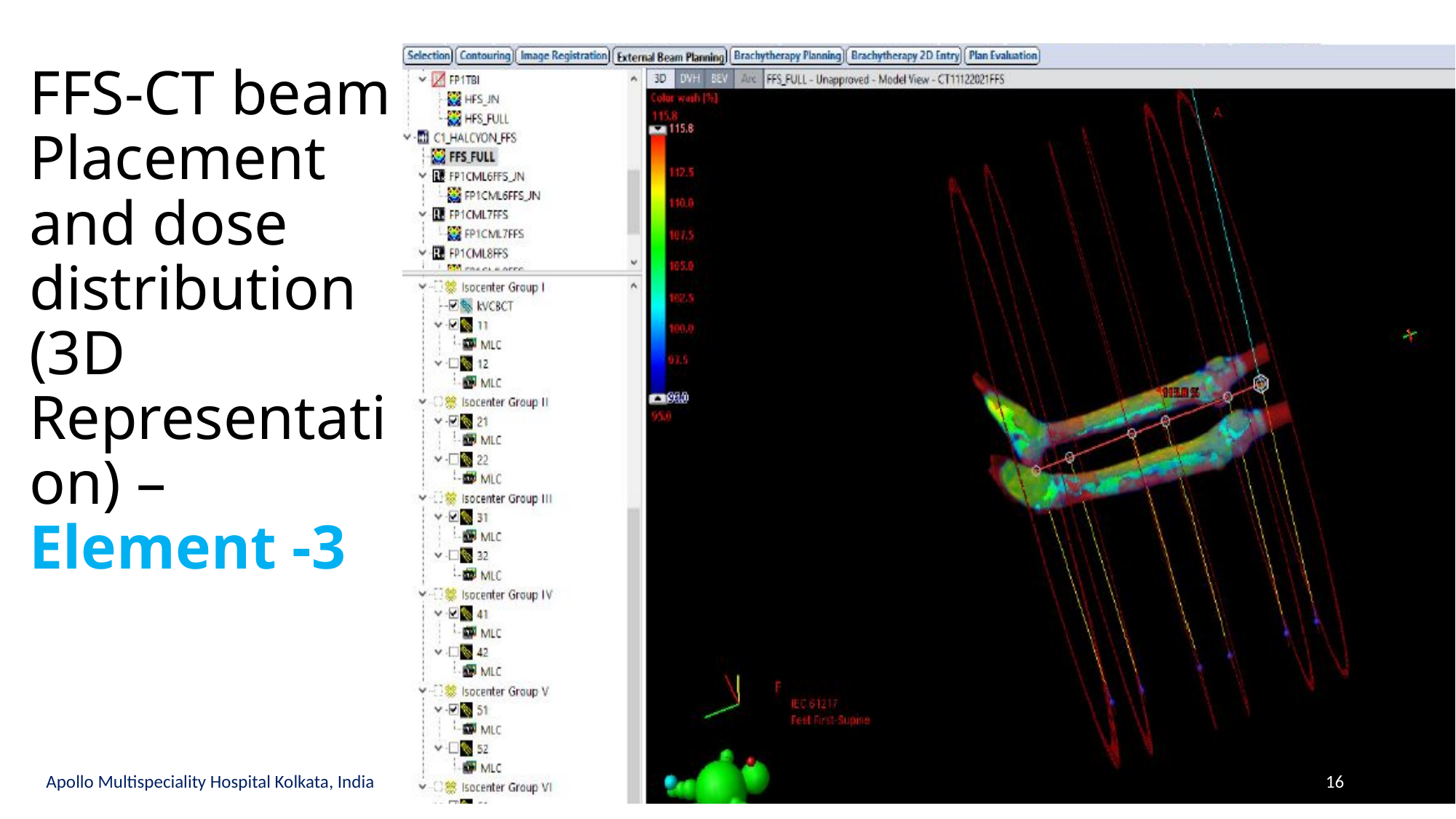

# FFS-CT beam Placement and dose distribution (3D Representation) – Element -3
Apollo Multispeciality Hospital Kolkata, India
16

## Slide 17
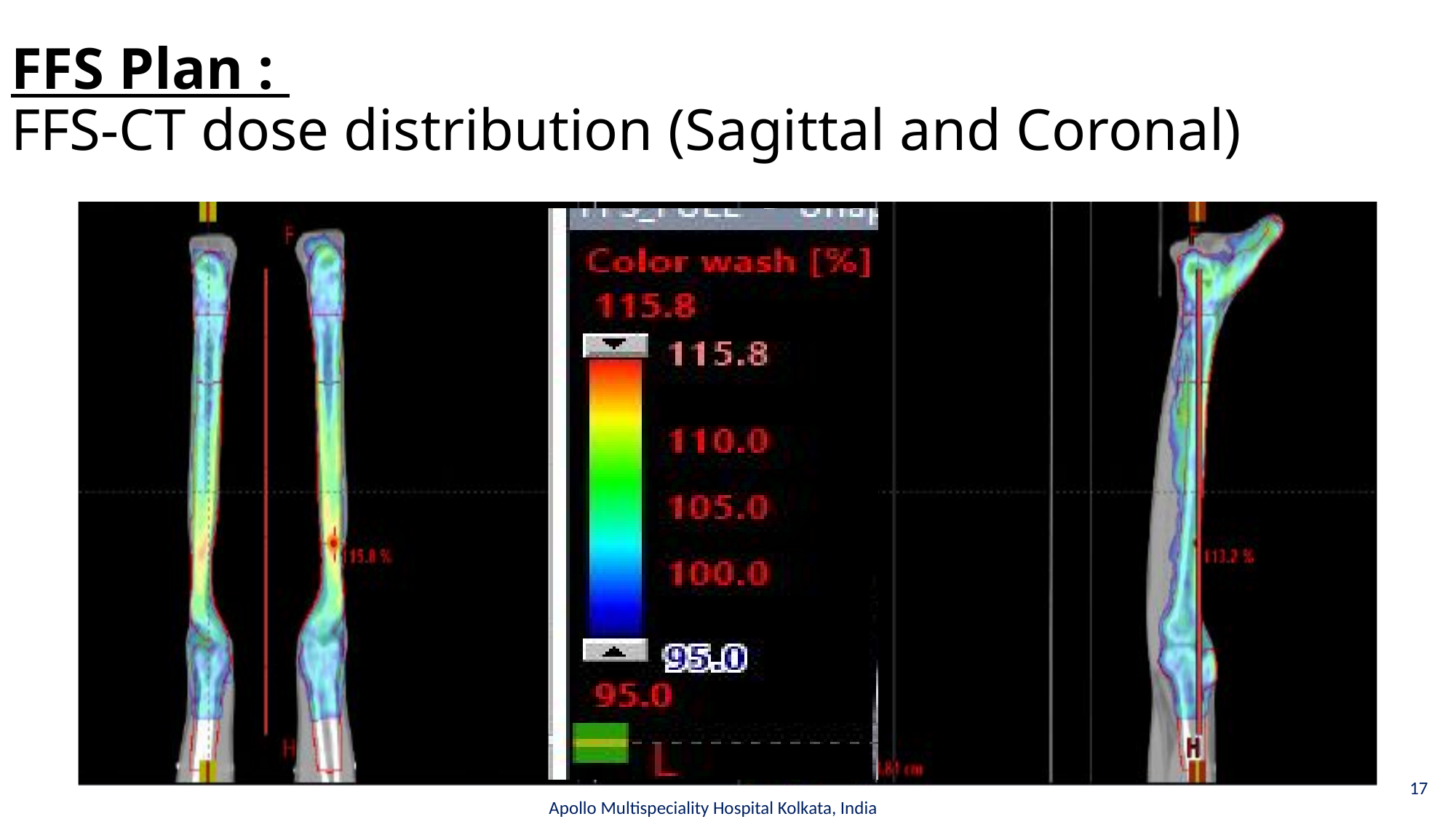

# FFS Plan : FFS-CT dose distribution (Sagittal and Coronal)
17
Apollo Multispeciality Hospital Kolkata, India

## Slide 18
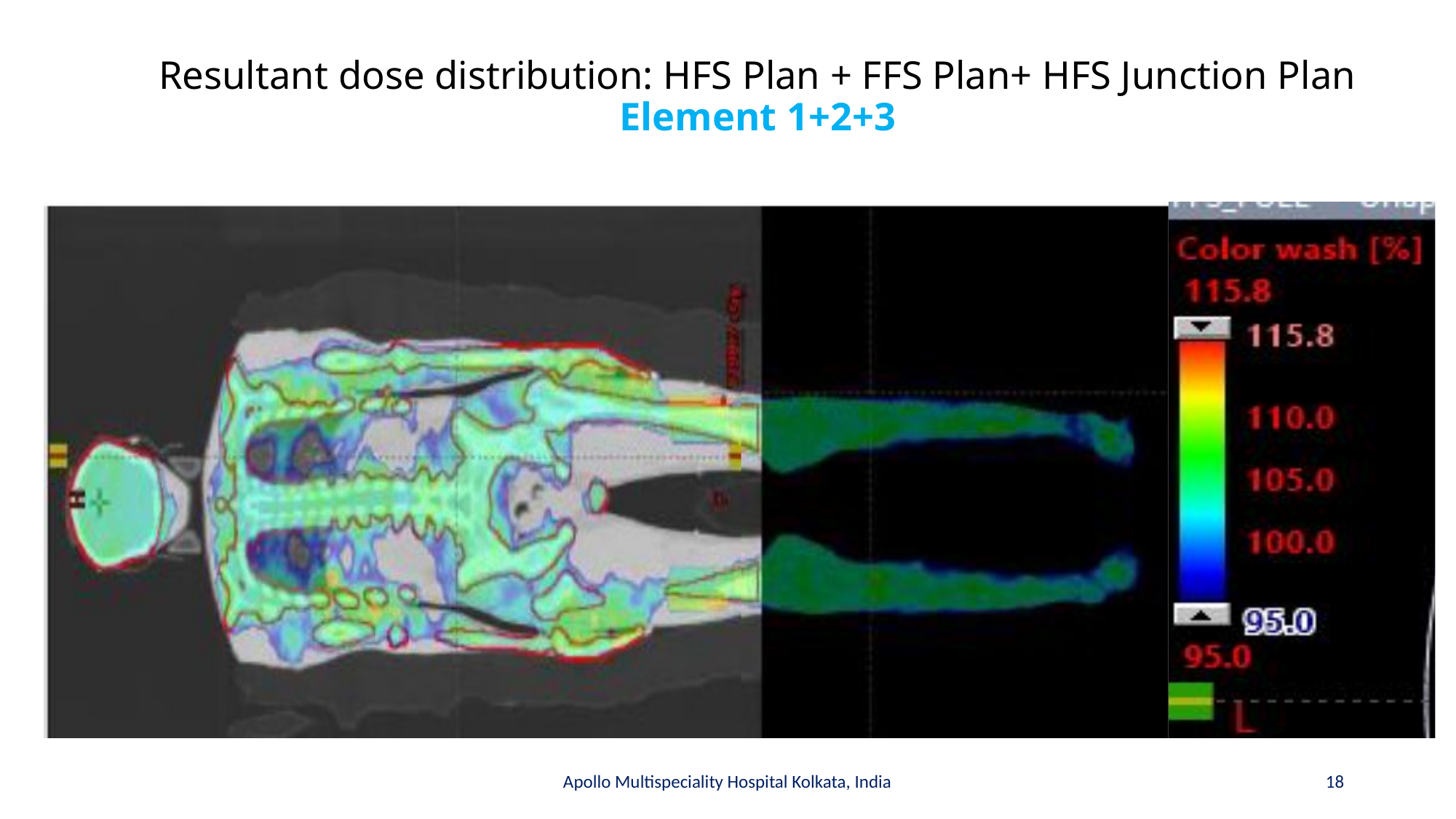

# Resultant dose distribution: HFS Plan + FFS Plan+ HFS Junction Plan Element 1+2+3
Apollo Multispeciality Hospital Kolkata, India
18

## Slide 19
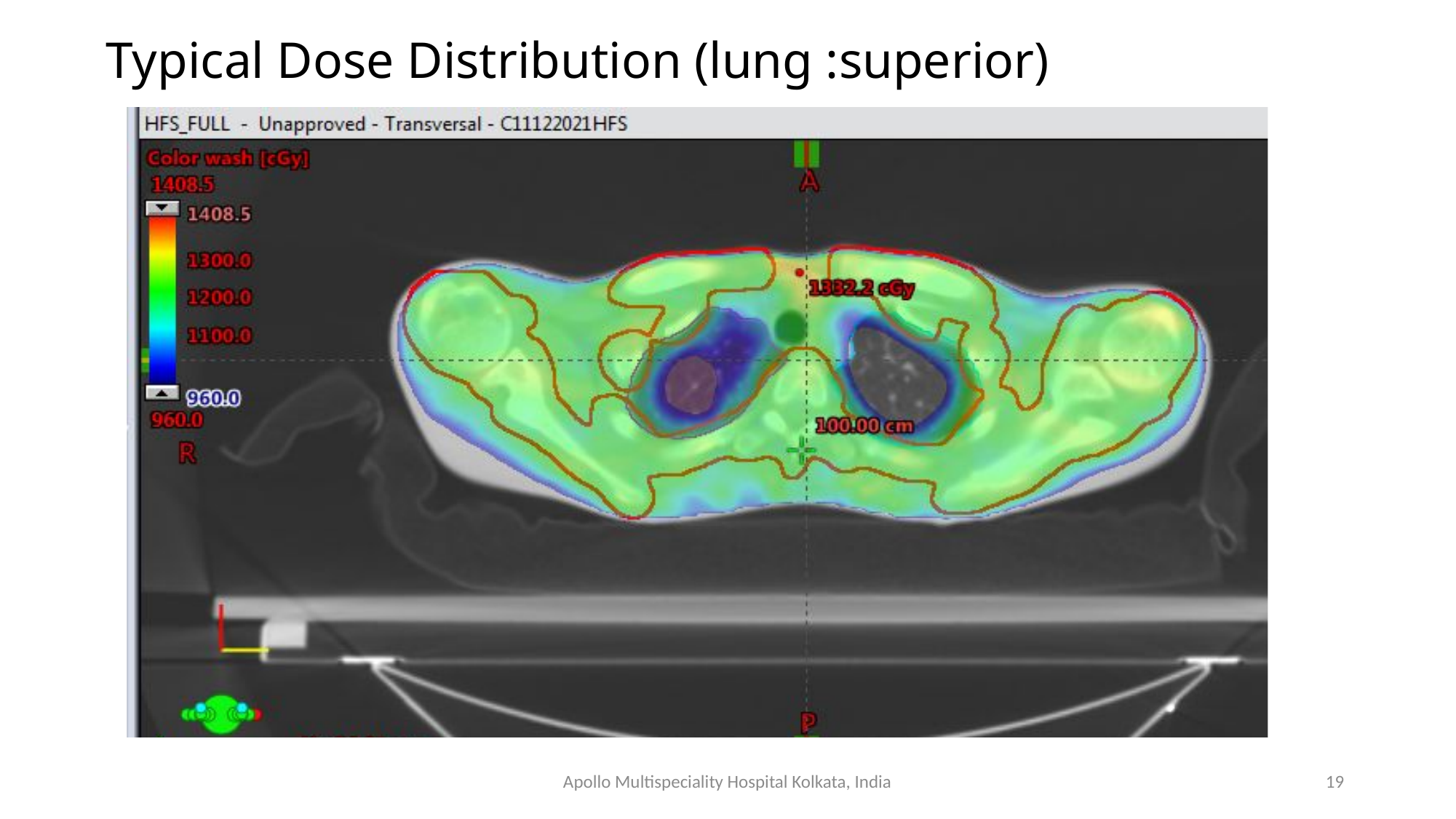

# Typical Dose Distribution (lung :superior)
Apollo Multispeciality Hospital Kolkata, India
19

## Slide 20
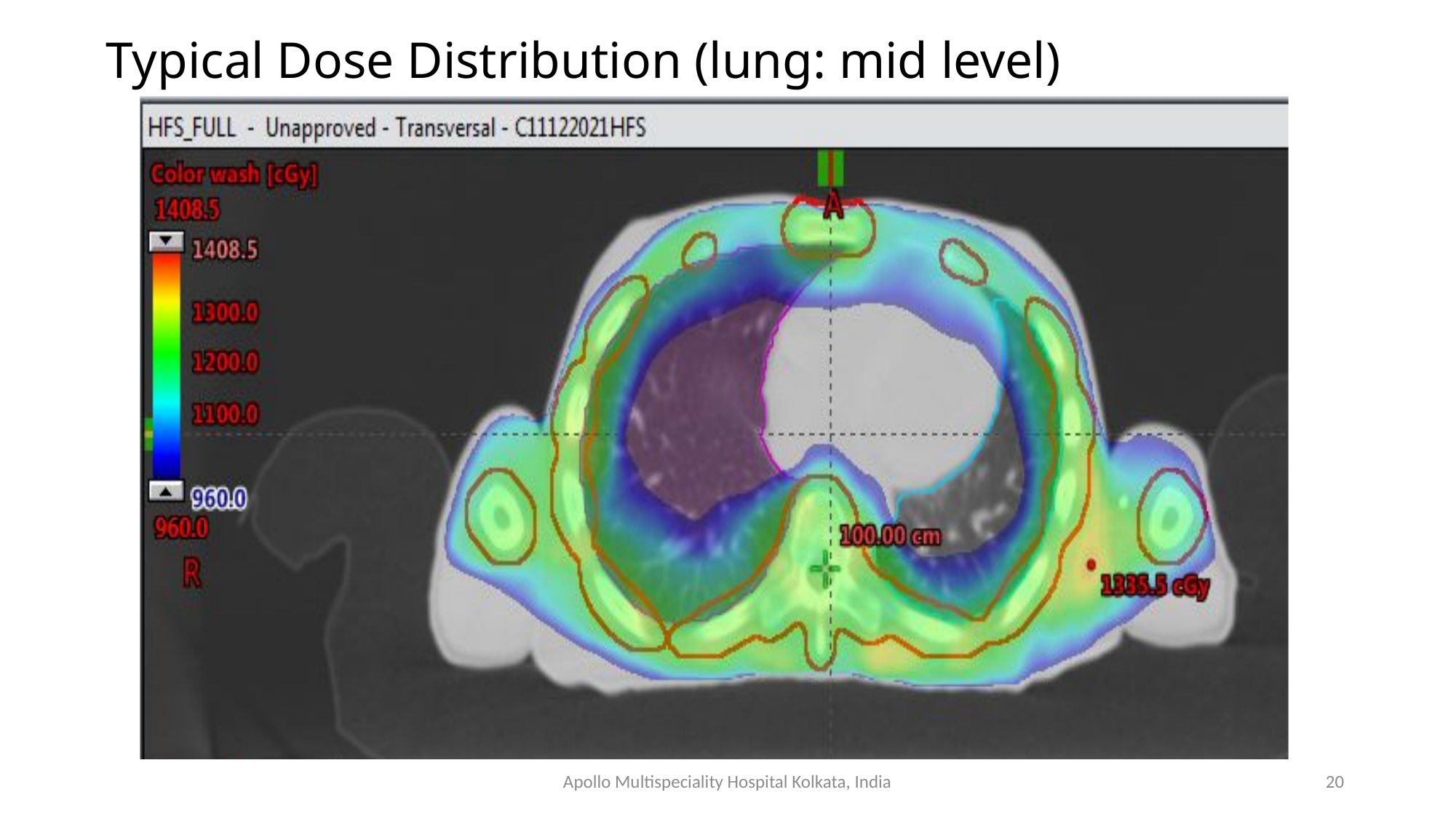

# Typical Dose Distribution (lung: mid level)
Apollo Multispeciality Hospital Kolkata, India
20

## Slide 21
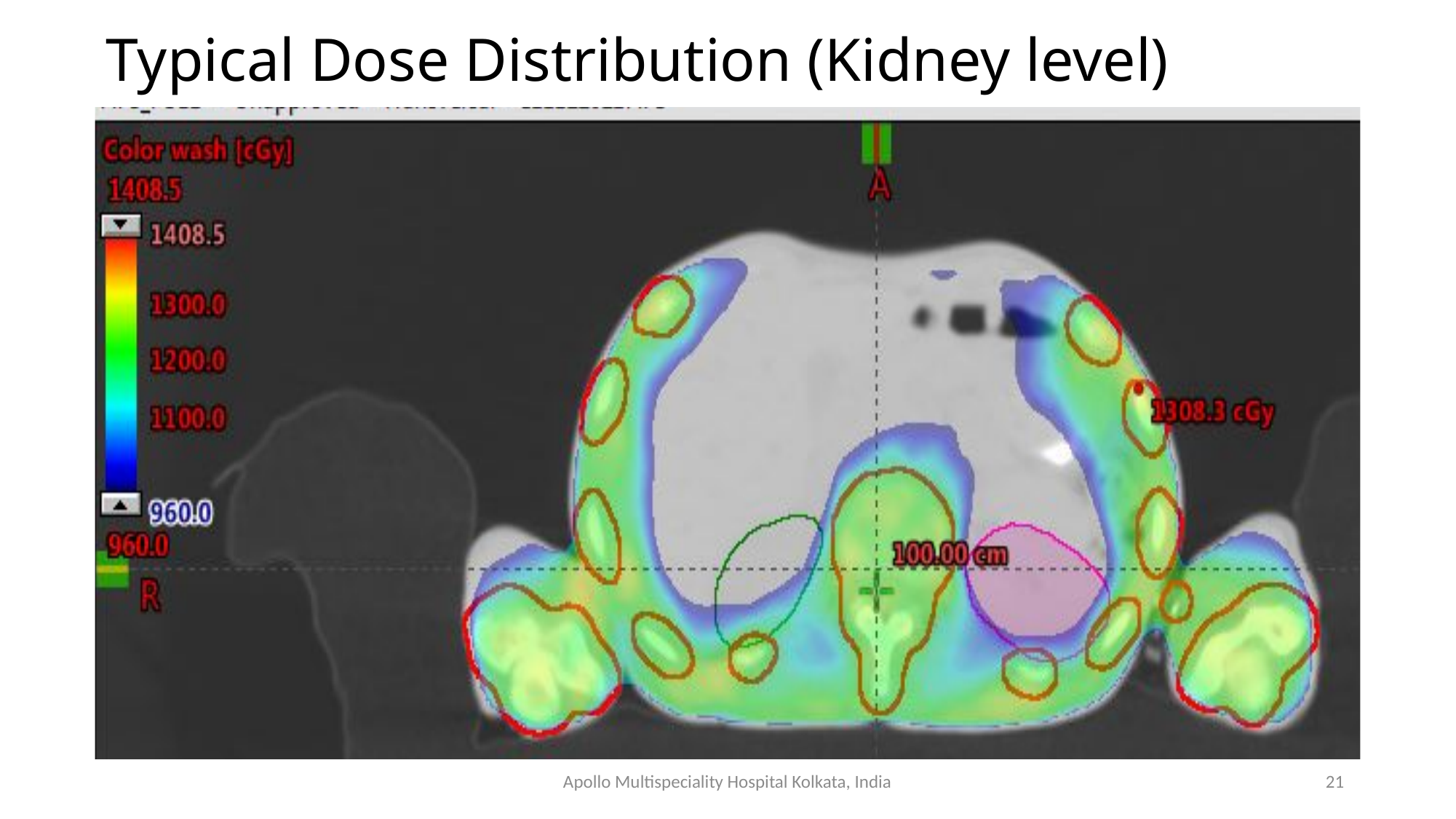

# Typical Dose Distribution (Kidney level)
Apollo Multispeciality Hospital Kolkata, India
21
